# Longitudinal study of the interplay between the skin barrier and facial microbiome over 1 year

**DOI:** 10.3389/fmicb.2023.1298632

**Published:** 2023-11-16

**Authors:** Jung Yeon Seo, Seung Won You, Ki-Nam Gu, Hanji Kim, Joong-Gon Shin, Sangseob Leem, Bo Kyoung Hwang, Yunkwan Kim, Nae Gyu Kang

**Affiliations:** Research and Innovation Center, R&D Center, LG H&H Co., Ltd., Seoul, Republic of Korea

**Keywords:** skin microbiome, TEWL, skin biophysical characteristics, *Cutibacterium*, *Corynebacterium*, *Staphylococcus*, *Neisseriaceae*, *Streptococcus*

## Abstract

Skin is a diverse ecosystem that provides a habitat for microorganisms. The skin condition and the skin microbiome interact each other under diverse environmental conditions. This study was conducted on 10 study participants for a one-year, from September 2020 to August 2021, to investigate the variability of skin microbiome and skin biophysical parameters [TEWL, hydration, and elasticity (R5)] according to season, and to understand the interplay between skin microbiome and skin characteristics. We identified that *Cutibacterium*, *Corynebacterium*, *Staphyloccocus*, unclassified genus within *Neisseriaceae*, and *Streptococcus* were major skin microbial taxa at the genus level, and fluctuated with the seasons. *Cutibacterium* was more abundant in winter, while *Corynebacterium*, *Staphylococcus*, and *Streptococcus* were more abundant in summer. Notably, *Cutibacterium* and skin barrier parameter, TEWL, exhibited a co-decreasing pattern from winter to summer and showed a significant association between *Cutibacterium* and TEWL. Furthermore, functional profiling using KEGG provided clues on the impact of *Cutibacterium* on the host skin barrier. This study enhances our understanding of the skin microbiome and its interplay with skin characteristics and highlights the importance of seasonal dynamics in shaping skin microbial composition.

## Introduction

The skin is a primary physical barrier against the invasion of pathogens, and also is a diverse ecosystem that can be a habitat for microorganisms including bacteria and fungi (Chiller et al., 2001; Fredricks, 2001). The ecology of the skin is varied topographically and influenced by various elements including environment and host factors (Fierer et al., 2008; Grice et al., 2009; Isler et al., 2023). The host’s characteristics, such as age and sex, contribute to the diversity of the skin microbiome (Somerville, 1969; Grice and Segre, 2011). On the other hand, the environmental factors such as temperature and humidity, have been reported as stimuli of the growth of skin microbiome (Duncan et al., 1969). The ultraviolet radiation stimulates the overproduction of oil glands and thickens the outermost layer of skin (Pearse et al., 1987; Lesnik et al., 1992), which may lead to the growth of lipophilic microorganisms (Dréno et al., 2018; Kobayashi et al., 2019).

Recent studies have revealed that the skin microbiome plays an important role in maintaining skin health under specific environmental conditions (Gallo and Nakatsuji, 2011; Byrd et al., 2018). The commensal skin microbiome produces substances similar to antimicrobial peptides that contribute to the development and maintenance of the skin’s immune system (Cogen et al., 2010). The disruption of the balance between commensal and pathogenic microorganisms can lead to the onset of skin diseases (Fitz-Gibbon et al., 2013; Liu et al., 2020). Additionally, the barrier function of the skin can be influenced by skin microbiome that produces metabolites activating aryl hydrocarbon receptor, which promotes epithelial differentiation and integrity (Uberoi et al., 2021). Typically, studies investigating the associations between microbial community and specific skin phenotypes divide subjects into case and control groups. However, even within these groups, individual physiological variations persist, potentially impacting research outcomes. Intra-individual microbiome assessments are therefore useful for examining substantial associations between microbial variation and the host’s skin condition by controlling for individual intrinsic traits. A longitudinal examination of the skin microbiome may reveal the interplay between microbial dynamics and skin characteristics.

To investigate compositional variation of skin microbiome, several studies analyzed bacterial and fungal communities of human skin over various time ranges. The longitudinal studies conducted within 6 months demonstrated that composition and diversity of skin microbiome can vary temporally depending on the skin sites and subjects (Grice et al., 2009; Flores et al., 2014). On the other hand, studies conducted over a longer period of time (1–2 years), which are the most extended time scale for investigation of skin microbiome, showed that the compositional features of the skin microbiome tend to remain relatively stable (Oh et al., 2016; Hillebrand et al., 2021; Schmid et al., 2022). However, although several longitudinal studies on the skin microbiome have been performed, understanding of the complex relationship between skin microbiome and skin characteristics is still limited. This is largely due to the fact that most long-term studies on skin characteristics have been conducted independently of those on the skin microbiome (Youn et al., 2005; Nam et al., 2015; Dolečková et al., 2021), which has resulted in a lack of comprehensive understanding of their interplay.

To investigate the interactions between skin microbiome and skin characteristics, we conducted a longitudinal study tracking changes in the facial skin microbiome and various skin biophysical parameters, over a period of 1 year. We collected microbiome data at weekly intervals with a higher frequency compared to previous researches, enabling the capture of detailed changes in the microbial community and mitigating potential biases in microbiome sampling. Additionally, the skin characteristics of subjects were collected at monthly intervals simultaneously. Our study aims to offer a more comprehensive understanding of longitudinal alterations in microbiome composition and skin properties. It may help understand the interplay between these two traits and estimate the impact of microbial changes.

## Materials and methods

### Study participants

Participants with the following characteristics were excluded: (1) those who used systemic or topical antibiotics within 3 months prior to the study, (2) those who had cutaneous disease on the skin, and (3) those who had sensitive skin. A total of 10 healthy Korean participants (7 males and 3 females) voluntarily contributed in study. The mean age of the participants was 32.4 years. During the study period (12 months, from September 2020 to August 2021), all study participants wore a mask at least 8 h a day due to COVID-19. Facial skin microbiome samples were collected by having subjects swab their left cheek once a week using a Copan swab 480 CE (Copan, Brescia, Italy), following an infographic-guided procedure: (1) sampling right after waking up and before washing face, (2) rubbing the left cheek for 1 min. The use of cosmetics was prohibited at least 8 h before skin microbiome sample collection. All participants were required to wear a mask under routine office work conditions and to maintain cosmetic and hygiene routines as consistently as possible. All samples were stored at −80°C until further processing. Three facial skin biophysical parameters of study participants were evaluated once a month with several measurements as follows: Skin surface TEWL was measured using Tewameter® TM 300 (Courage and Khazaka GmbH, Cologne, Germany) and was expressed in grams per square meter per hour (g/m^2/h) (Gardien et al., 2016). Skin hydration was calculated using Corneomter CM 825 (Courage and Khazaka GmbH, Cologne, Germany). Changes in the capacitance of the stratum corneum were measured and were expressed in arbitrary units (CM) (Dąbrowska and Nowak, 2021). Skin surface elasticity was quantified using Cutometer MPA 580 (Courage and Khazaka GmbH, Cologne, Germany). R5 [immediate retraction (Ur)/immediate distension (Ue)], representing the net elasticity of the skin was used as elasticity index (Ohshima et al., 2013). This study was approved by the institutional review board at the LG H&H Research Center (Seoul, South Korea) and all study participants provided an institutional review board-approved written consent form (No. LGHH-20201210-AB-03).

### Skin microbiome sequencing

Genomic DNA was extracted from swab sample using the QIAamp mini kit (QIAGEN, Hilden, Germany). Quantity of extracted DNA was measured with a Qubit 4 Fluorometer (Invitrogen, Massachusetts, USA). 16S rRNA gene amplicon libraries were constructed following the instructions of Illumina’s 16S rRNA metagenomics sequencing library preparations with some adaptation (Illumina, 2014) as follows: (1) locus specific amplification with two specific primers for v3- v4 variable regions of 16S rRNA gene, 341F (5′ - CCTACGGGNGGCWGCAG - 3′) and 805R (5′ - GACTACHVGGGTATCTAATCC - 3′), (2) purification of the amplicon with AMPure XP beads (Beckman Coulter, Krefeld, Germany), (3) amplification for sample indexing with Nextera XT Index kit (Illumina, California, USA), (4) purification of the amplicon with AMPure XP beads (Beckman Coulter, Krefeld, Germany), (5) validation of constructed library quantity and quality (size and integrity) with Qubit 4 Fluorometer and 2100 Bioanalyzer (Agilent, California, USA), respectively. Final library of each sample was then normalized and pooled. Pooled final library was loaded into MiSeq Reagent Kit v3 (600-cycle) (Illumina) and was sequenced on an Illumina MiSeq with 2 × 300 bp paired-end read chemistry (Illumina) (Fadrosh et al., 2014). Quality of raw sequence reads were checked using the FastQC (Babraham Institute, Cambridge, UK) and Sequencing Analysis Viewer (Illumina).

### 16S rRNA gene sequences taxonomy classification

Demultiplexing of sequence reads was conducted by Illumina Miseq Reporter Software automatically. Subsequent pre-processing and clustering of demultiplexed paired-end sequence reads to obtain the clean amplicon sequence variants were processed by Quantitative Insights into Microbial Ecology 2 pipeline (2021.2.0) (Bolyen et al., 2019). In detail, the primer sequences used to amplify the v3- v4 variable regions of 16S rRNA gene were trimmed using q2-cutadapt plugin based on Cutadapt (2021.2.0) (Kechin et al., 2017). After trimming, the denoising of paired-end sequence reads, removal of chimeric sequences, and read fusion were conducted through q2-dada2 plugin based on DADA2 (2021.2.0) (Callahan et al., 2016). With the scikit-learn naïve-bayes model based pre-trained taxonomy classifier on GreenGene 13.8 99% reference database, all processed reads were matched to proper microbiome taxonomy. Further statistical analysis for the facial microbiome in this study was conducted on genus level and skin microbiome samples with low sequencing quality (read count ≦ 12,000, passing filter ≦ 80%) were excluded.

### Statistical analysis

All statistical analyses were performed using R version 3.6.0. Relative abundance of microbiome was obtained by dividing the number of reads of one genus by the number of reads of all genus per subject. In order to obtain robust data, the mean value of the relative abundance of the microbiome within each participant per month (each participant was sampled per week, accumulating 4–5 samples in 1 month) was used, and four seasons were classified according to the month (spring: March, April, May; summer: June, July, August; fall: September, October, November; winter: December, January, February). The objective of the study was to observe changes in the overall microbiome. Therefore, the major five genera were selected based on their average relative abundance, which was greater than 1% in at least one-third of the samples. To compare the relative abundance of major taxa between summer and winter, Wilcoxon rank sum test was used.

Shannon diversity was calculated to examine microbiome evenness and richness of each sample (α-diversity) (Olszewski, 2004). Jensen-Shannon distance was also calculated for measuring dissimilarity between microbiome compositions of each sample (β-diversity) (Lin, 1991). To assess the statistical significance of β-diversity, permutational analysis of variance was used on the Jensen-Shannon distance matrices with 999 permutations in vegan 2.5–7 package in R (Anderson, 2001).

Normalization through Z-score transformation was conducted on measured skin parameters and the relative abundance of *Cutibacterium, Corynebacterium*, *Staphylococcus*, unclassified genus within *Neisseriaceae* (F), and *Streptococcus*. Canonical Correspondence Analysis (CCA) (Braak, 1986) was conducted to explain the dispersion of the microbiome communities with reference to factors including weather information, skin biophysical parameters and relationships between these factors. Jensen-Shannon distance matrix was used in microbiome 1.8.0 (Lahti and Shetty, 2017), vegan 2.5-7 (Oksanen et al., 2022), phyloseq 1.30.0 packages in R (McMurdie and Holmes, 2013). All statistical analyses were performed with R software (version 3.6.3.), and the ggplot2 package was used to visualize results (Wickham, 2009). To elucidate the association of skin biophysical parameters and the relative abundance of each five genera, linear regression analysis was conducted while adjusting for individual differences. Linear regression analysis adjusted for weather information (temperature, humidity: downloaded from Korea Meteorological Administration) (Supplementary Figure S1) was also performed. We considered *p*-value significant if less than 0.05. *p*-values were adjusted using the false discovery rate correction method by Benjamini-Hochberg procedure for multiple testing. Kruskal-Wallis test was used to test for significant differences in α-diversity between four season groups. Pairwise Wilcoxon rank sum test was used to compare the relative abundance of each major genus or skin biophysical parameters between pairs of seasons.

### Functional profiling of skin microbiome

Functional profiling of the skin microbiome was conducted using q2-picrust2 (2021.2), which is based on PICRUSt2 (Douglas et al., 2020). PICRUSt2, tool for predicting functional abundances based on marker gene sequences, predicted the enriched pathway by inferring the functional profile of the facial skin microbiome based on Kyoto Encyclopedia of Genes and Genomes (KEGG) orthology gene family database (Kanehisa et al., 2004, 2012). For the purpose of identifying the pathway that can explain the difference between groups, the process was applied: comparing the KEGG orthology annotation results from PICRUSt analysis between the groups using the Kruskal-Wallis test for whole seasons, and using Wilcoxon rank-sum test for comparing summer and winter while adjusting individual factors.

## Results

### Seasonal variation in skin microbiome composition and diversity

**Figure 1:**
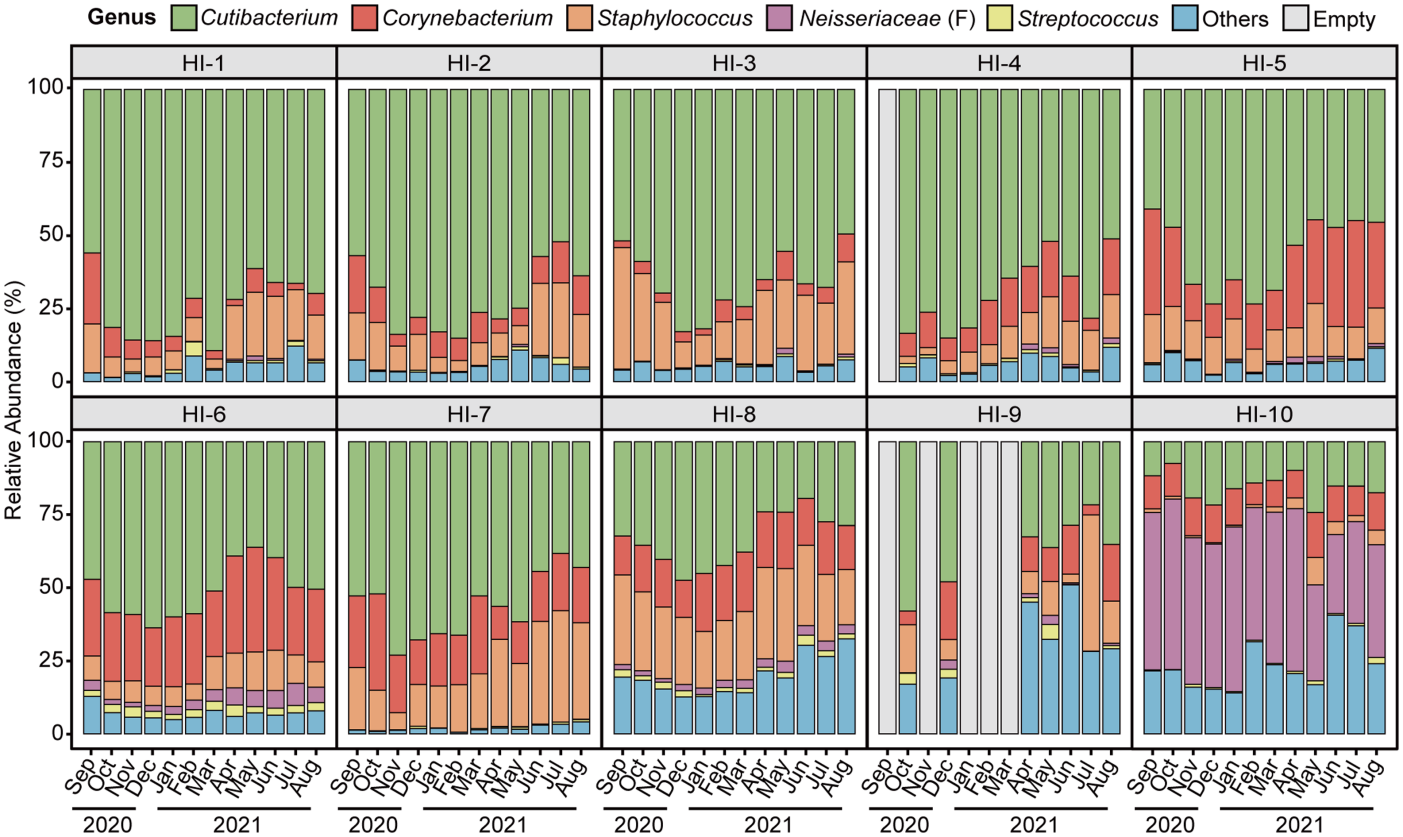
The skin microbiome composition of study participants by month of the year. Average per month the relative abundance of major skin microbiome genera (*Cutibacterium*, *Corynebacterium*, *Staphylococcus*, unclassified genus within *Neisseriaceae* (classified only at the family level), and *Streptococcus*) of study participants. Microbial taxa which are found in less than one-third of study participants and having low abundance taxa (<1%) are grouped as ‘others’. HI, healthy individual.

We collected a total of 358 skin microbiome data from ten participants by weekly swab sampling and measured individual skin characteristics monthly. The workflow of this study is presented in Supplementary Figure S2 and a summary of the skin biophysical parameters of the study participants is presented in Supplementary Table S1. The skin microbiome compositions at the genus level of study participants on a monthly basis for 1 year are shown in Figure 1. The microbiome composition varied longitudinally, with each individual exhibiting a distinct microbial profile. Among various microbiome genera, *Cutibacterium* was the most dominant genus among 9 participants, with an average relative abundance of from 34 to 74% per individual. Additionally, one individual showed unclassified genus within *Neisseriaceae* (classified only at the family level) is the most dominant taxa.

**Figure 2:**
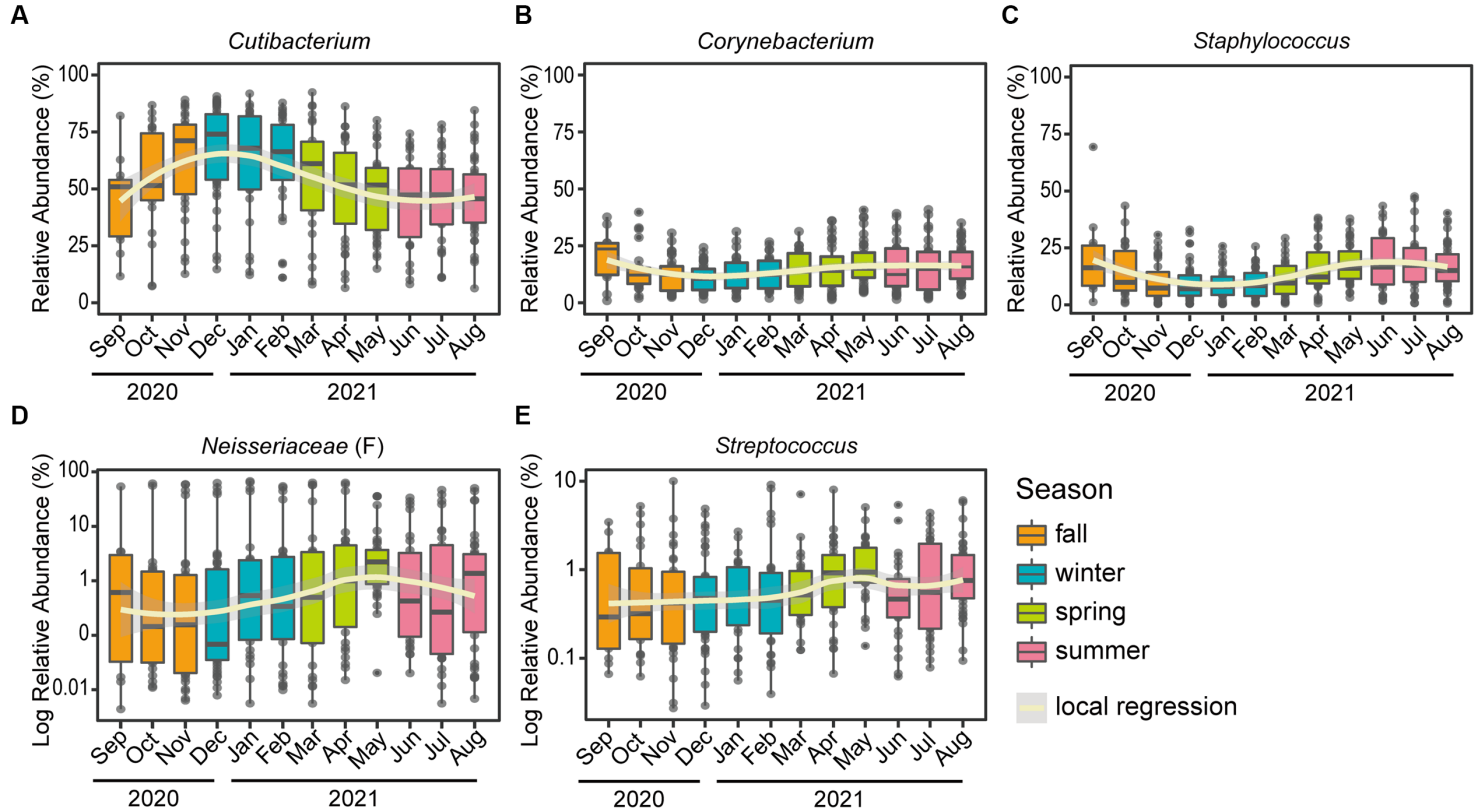
Seasonal variation of the top five skin microbiome at the genus level. Boxplots of the relative abundance for the five major genera: **(A)** *Cutibacterium*, **(B)** *Corynebacterium*, **(C)** *Staphylococcus*, **(D)** unclassified genus within *Neisseriaceae* (classified only at the family level) and **(E)** *Streptococcus*. A polynomial local regression line was added to boxplots to enhance visualization of the trend. Relative abundances of panel **(D)** unclassified genus within *Neisseriaceae* and **(E)** *Streptococcus* were converted to a logarithmic scale (log10), to increase legibility.

At the genus level, *Cutibacterium*, *Corynebacterium*, *Staphylococcus*, unclassified genus within *Neisseriaceae* (F) and *Streptococcus* were major skin microbial taxa, which accounted for approximately 90% of the skin microbial community composition, and exhibited seasonal fluctuations (Figures 1, 2). In particular, the relative abundance of *Cutibacterium* was statistically significantly more abundant in winter than in summer (63.97 and 45.50%, respectively, *p*-value = 2.5E-10), while *Corynebacterium*, *Staphylococcus*, and *Streptococcus* were more abundant in summer than in winter (*p*-value <0.05).

**Figure 3:**
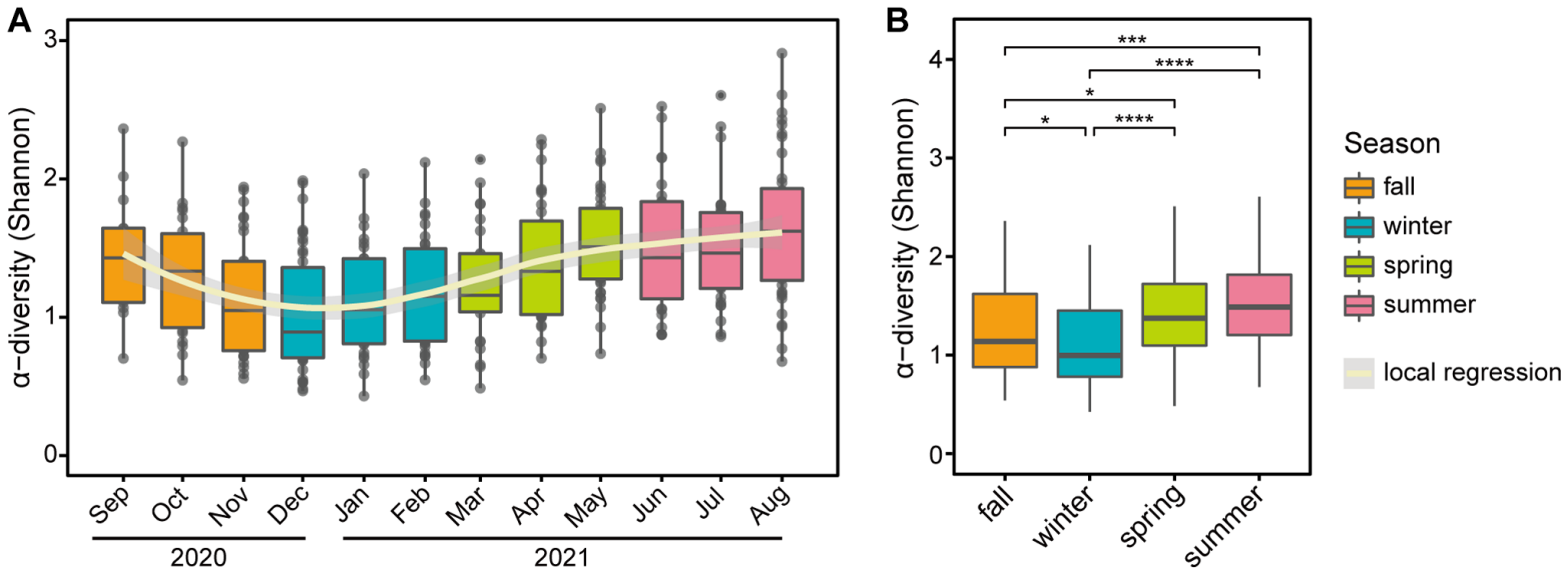
Seasonal variation of α-diversity at the genus level. Boxplots of α-diversity based on panel **(A)** month and **(B)** season. In panel **(A)**, a polynomial local regression line was added to boxplots to enhance trend visualization. In panel **(B)**, significant differences in pairwise comparison are marked with stars (*p-value <0.05, **p-value < 0.01, ***p-value < 0.001, and ****p-value < 0.0001).

The α-diversity of the microbial community exhibited seasonal variation (Figure 3A), with significant differences observed between the four seasons (*p*-value = 1.6E-10, Figure 3B). Notably, there were significant differences between most pairs of seasons (*p*-value <0.05) except for comparing spring 2021 and summer 2021 (Figure 3B). The β-diversity based on Jensen-Shannon distance plots according to season, month, and individuals are presented in Supplementary Figure S3. To examine skin microbiome composition varied longitudinally, a permutational analysis of variance (PERMANOVA) was performed by season and month, respectively (Supplementary Tables S2, S3). The results of the PERMANOVA analysis showed that there were significant differences in the microbiome compositions among the four seasons for all pairwise comparisons, except for the comparison between fall 2020 and winter 2021 (*p*-value <0.05; Supplementary Table S2). Furthermore, the distance within seasons is significantly closer than the distance between seasons, indicating the clustering of microbial communities by season (Wilcoxon rank sum test *p*-value <2.2E-16). Also, the microbiome compositions were clearly separated by each individual (*p*-value <0.05) (Supplementary Table S4).

### Association analysis of skin barrier parameters and the skin microbiome

**Figure 4:**
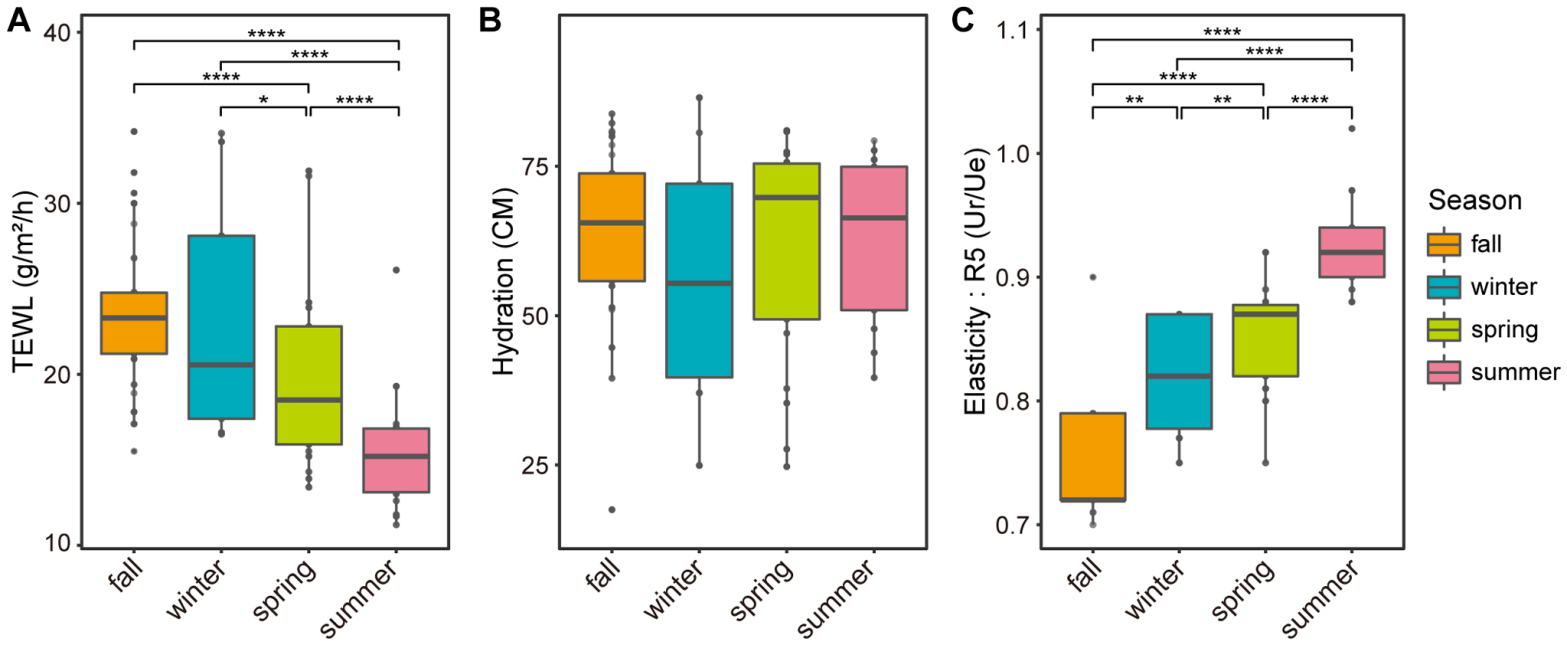
Boxplot of skin biophysical parameters based on the season. Boxplots of panel **(A)** skin surface transepidermal water loss, **(B)** skin hydration, and **(C)** skin surface elasticity (R5, Ur/Ue) based on the season. Ur: immediate retraction, Ue: immediate distension. Significant differences in pairwise comparison are marked with stars (*p-value < 0.05, **p-value < 0.01, ***p-value < 0.001, and ****p-value < 0.0001).

**Figure 5:**
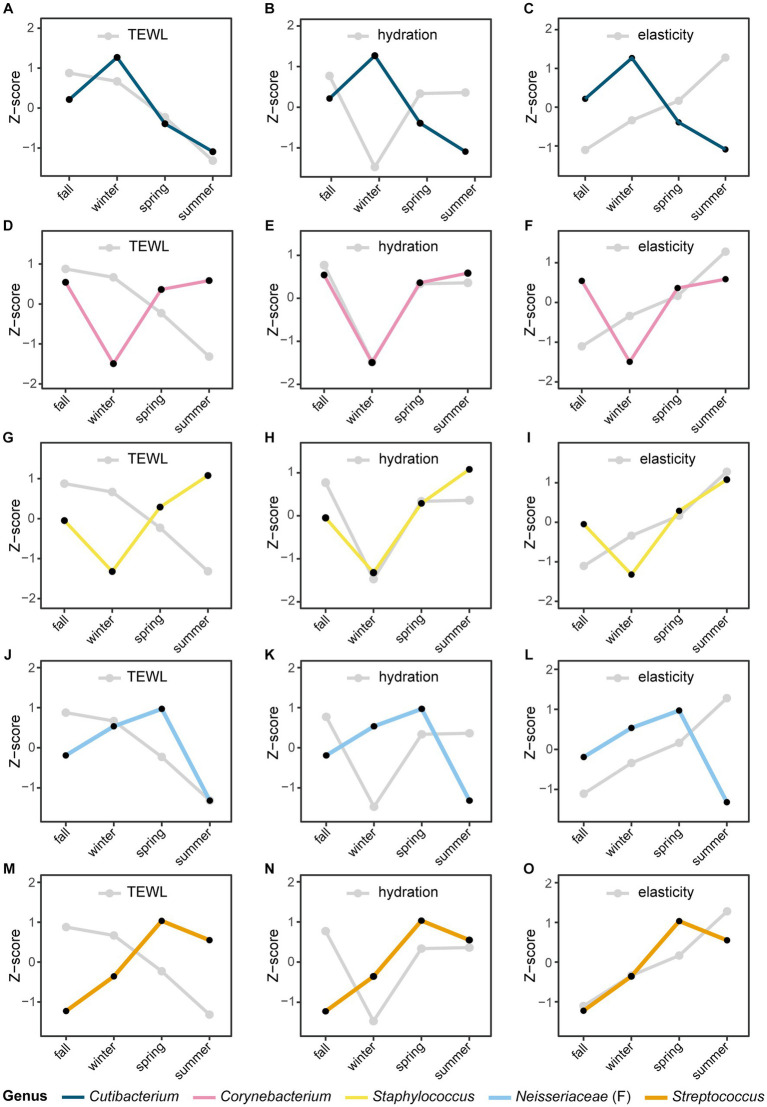
Seasonal variation of the microbial taxa and skin biophysical parameters. The longitudinal movement of the relative abundance of panels **(A–C)** *Cutibacterium*, **(D–F)** *Corynebacterium*, **(G–I)** *Staphylococcus*, **(J–L)** *Neisseriaceae* (classified only at the family level), and **(M–O)** *Streptococcus* at the genus level were compared with skin biophysical parameters [**(A,D,G,J,M)**: TEWL, **(B,E,H,K,N)**: skin hydration, **(C,F,I,L,O)**: skin elasticity]. The relative abundance of microbial taxa and skin biophysical parameters were normalized to the *Z*-score; each point represents the mean value for each season.

Seasonal variations were also observed in skin biophysical parameters, including TEWL, hydration, and elasticity (Figure 4; Supplementary Table S1). Especially, TEWL, which is a measure of skin barrier function, was significantly higher in the winter than in the summer (22.33 and 15.08, respectively, *p*-value = 5.0E-05). No significant differences in hydration were found between seasons, but elasticity showed significant differences in all pairs of seasons (Figures 4B,C). We noted longitudinal variations in both the five major microbial taxa and skin biophysical parameters, with remarkable differences between the two seasons (Figure 5). Notably, the abundance of *Cutibacterium* and TEWL exhibited a co-decreasing pattern from winter to summer and while the abundance of *Corynebacterium* and *Staphylococcus* showed the opposite pattern to TEWL (Figures 5A,D,G). In terms of hydration, there were coordinated patterns with *Corynebacterium* and *Staphylococcus* across the seasons, but an opposite pattern was observed in *Cutibacterium* with regards to hydration (Figures 5B,E,H). Elasticity showed co-increasing patterns with *Staphylococcus* and *Streptococcus* from winter to summer, but conversely, the opposite pattern was observed with *Cutibacterium* (Figures 5C,I,O).

The Canonical Correspondence Analysis (CCA) was used for investigating the correlation between skin microbiome composition, weather conditions, and skin biophysical parameters (Supplementary Figure S4). The total inertia was 0.94 and the constrained inertia was 0.18, of which 9.6% was explained by the CCA1 axis and 3.8% by CCA2 axis. The length of each arrow reflects the strength of the variable in explaining the observed dispersion of the microbiome. Specifically, TEWL and hydration had a substantial impact on the dispersion of the microbiome.

To investigate the association between skin parameters and the skin microbiome that changes with seasonal changes, linear regression analyses were conducted using the whole collected skin microbiome data and skin biophysical data in study period. As a result, TEWL was significantly associated with the relative abundance of *Cutibacterium*, *Corynebacterium*, and *Staphylococcus*, respectively (*p*-value <0.05) (Table 1). Elasticity (R5) was also significantly associated with the relative abundance of *Cutibacterium*. After adjusting with false discovery rate, only *Cutibacterium* was significantly associated with TEWL (false discovery rate-adjusted *p*-value = 1.5E-05). *Cutibacterium* was also significantly associated with TEWL after adjusting for individual, temperature, and humidity (*p*-value = 0.02).

**Table 1:** Association analysis of the relative abundance of microbial taxa at the genus level with skin biophysical parameters.

Skin biophysical parameters	*Cutibacterium*			*Corynebacterium*			*Staphylococcus*		
	Beta	*P* ^a^	*P*_corr_ ^b^	beta	*P* ^a^	*P*_corr_ ^b^	beta	*P* ^a^	*P*_corr_ ^b^
TEWL	0.14	**9.7 × 10** ^**−7**^	**1.5 × 10** ^**−5**^	−0.14	**8.8 × 10** ^**−3**^	ns	−0.09	**0.02**	ns
Hydration	–	ns	ns	–	ns	ns	–	ns	ns
Elasticity (R5)	−9.6 × 10^−4^	**0.04**	ns	–	ns	ns	–	ns	ns

Significant associations are shown in bold face (*p*-value < 0.05).

ns, not significant; TEWL, transepidermal water loss.

a *p*-value of linear regression analysis by adjusting for individuals as covariates.

b *p*-value after adjusted with false discovery rate.

### Functional analysis of skin microbiome according to season

To identify functional differences in the skin microbiome across different seasons, we conducted a predictive analysis of metagenome function by inferring enriched pathways based on sequences using PICRUSt2, which is based on the Kyoto Encyclopedia of Genes and Genomes (KEGG) database. At the second level of analysis, among 40 pathways, 36 pathways showed significant differences between seasons (false discovery rate-adjusted *p*-value <0.05) (Supplementary Figure S5). In particular, 33 pathways showed noticeable differences between summer and winter (Supplementary Table S5). For instance, pathways related to cell motility, the circulatory system, aging, and signal transduction were enriched in summer (Supplementary Figure S5). At the third level of analysis, among 390 pathways, 264 pathways were significantly different between summer and winter. Notably, pathways related to glycosaminoglycan degradation, oxidative phosphorylation, and porphyrin and chlorophyll metabolism were enriched in winter (Supplementary Table S6).

## Discussion

Understanding the skin microbiome is important as it plays an essential role in maintaining skin’s homeostasis by protecting against pathogens or controlling the immune system (Schommer and Gallo, 2013; Kobayashi et al., 2019). The impact of external environmental factors on both the skin condition and skin microbiome has been extensively investigated in previous studies (Leeming et al., 1984; Grice and Segre, 2011; Kobayashi et al., 2019; Isler et al., 2023). Since temperature and humidity obviously fluctuate with the season (Supplementary Figure S1), it is important to investigate skin microbiome and skin biophysical parameters along with seasonal changes. However, most previous studies have not investigated skin conditions and skin microbiome together longitudinally. Therefore, we conducted a one-year longitudinal study on skin biophysical parameters and skin microbiome, simultaneously.

Previous studies suggested that the bacterial community of the skin remains stable for approximately 1–2 years. However, these studies performed sampling only once or twice a year, which was insufficient to observe the annual fluctuations of the microbial community (Flores et al., 2014; Hillebrand et al., 2021). In a recent longitudinal study conducted by Oh et al. (2016), the annual variation in the mycobiota of healthy individuals was examined by collecting samples once a month, revealing that the mycobiota remained relatively stable throughout the year. However, it should be noted that this study focused solely on fungi and did not investigate bacteria and skin characteristics. Although these findings provide valuable insight into mycobiota dynamics in healthy individuals, further research is needed to fully understand the complex interplay between the microbiome and skin health.

In our study, we collected skin microbiome samples once a week and skin biophysical parameters once a month, which allowed us to collect a large amount of data and ensure the accuracy of our study results to study the relationship between skin and skin microbiome. As a result, we discovered the composition of the skin microbiome fluctuated with the seasons, in particular, that observed in the major skin microbial taxa, including *Cutibacterium*, *Corynebacterium*, and *Staphylococcus*. Especially, the HI-10 participant exhibited a distinct most abundant taxon, an unclassified genus within *Neisseriaceae*, which has been reported as one of the dominant microbial taxa in human skin (Qiao et al., 2021). The β-diversity plot also showed a distinct cluster of HI-10 samples from the others. Functional studies should be conducted further to untangle the potential relationships between dominant taxa and human skin properties. The α-diversity showed seasonal variation and β-diversity analysis revealed that microbial communities were distinct by season. Moreover, the Jensen-Shannon distance within seasons is significantly closer than between seasons suggests that microbial communities undergo seasonal changes, highlighting the importance of seasonal dynamics in shaping skin microbial composition. Seasonal variations were also observed in skin biophysical parameters, including TEWL, hydration, and elasticity. The relative abundance of *Cutibacterium* and TEWL was significantly high in winter and low in summer. In particular, our study’s CCA and linear regression result provide clues that can explain the association between skin microbiome and skin biophysical characteristics. The results of CCA analysis demonstrate that TEWL and hydration have more impacts on microbiome dispersion, and the relative abundance of *Cutibacterium* is correlated with TEWL. Especially, there was a significant association between *Cutibacterium* and TEWL, even after adjusting for individual differences and weather indicating a dependent relationship.

The epidermal barrier function is known to vary depending on the season, and the difference in the integrity of the skin barrier is mainly caused by epidermal ceramide, which is a major component of the skin barrier (Coderch et al., 2003; Wei et al., 2016; Pappas et al., 2018). The level of epidermal ceramide is widely recognized to be regulated by various factors such as skin microbiome and skin disease (Zheng et al., 2022; Schachner et al., 2023). Several studies have demonstrated that the skin microbiome can have an impact on the physical barrier of the skin (Liu et al., 2020; Harris-Tryon and Grice, 2022). Considering previous studies, we found that the major taxa that fluctuated with the seasons, may regulate the epidermal ceramide levels and subsequently impact the skin barrier function. *S. epidermidis*, one of the *Staphylococcus* species, helps maintain skin barrier integrity through the secretion of sphingomyelinase, which is crucial for the production of ceramide (Zheng et al., 2022). *C. acnes* is known to account for more than 90% of *Cutibacterium* in human skin (Li et al., 2021) and can partially hydrolyze triglycerides in sebum which acts as a pathway for water diffusion into the epidermis (Riegels-Nielsen et al., 1989; Grice and Segre, 2011; Rocha and Bagatin, 2018; Kobayashi et al., 2019). In addition, we performed functional profiling of the skin microbiome across seasons using KEGG to interpret how the skin microbiome may affect skin barrier function. As a result, it was possible to partially interpret and infer the effect of *Cutibacterium*, which accounts for more than half of the total individual’s microbiome composition, on the host skin barrier. For instance, porphyrin metabolism was elevated in winter, which is known to be produced by *Cutibacterium acnes*, can induce oxidative stress and cause skin inflammation (Barnard et al., 2020; Spittaels et al., 2021; Stødkilde et al., 2022). Although the resulting pathways operate at the cellular level and the functional relationship is not clear, the increase in glycosaminoglycan degradation pathway during winter may play an important role in the elevated loss of water content in the epidermal skin. Hyaluronic acid, one of the extracellular matrix components in the epidermis and dermis, is a type of glycosaminoglycan and is known to be degraded by hyaluronate lyase produced by *C. acnes* (Nazipi et al., 2017; Li et al., 2021; Mayslich et al., 2021). Moreover, a previous anti-acne treatment study has reported improvement in skin barrier function (reduced TEWL) and simultaneously the relative abundance of *Cutibacterium* (formerly *Propionibacterium*) tended to decrease (Shao et al., 2023). These results can partially explain the impact of the skin microbiome on skin characteristic such as skin barrier function.

Our study has the advantage of longitudinally observing variations by concurrently collecting skin microbiome and skin biophysical characteristics. This approach enables us to explore the intricate association between skin microbiome and host skin characteristics. Nonetheless, it is imperative to acknowledge the limitations of our study. The composition of the microbiome can be influenced by various factors such as the surrounding environment and host’s lifestyle (Prescott et al., 2017; Moitinho-Silva et al., 2021). While information about environments regarding humidity, and temperature, is available, details about the host’s lifestyle are lacking in our study. Since we consistently collected skin microbial samples and conducted skin measurements in the same facial area, we consider that the aspects related to personal hygiene habits such as mask-wearing and cosmetics were controlled. Specifically, throughout the entire duration of the study, mask-wearing was required and their cosmetic and hygiene routines were maintained consistently. However, to investigate a more systemic relationship between host and skin ecology, we propose the collection of survey data about host lifestyle information. By considering such factors, we anticipate a more comprehensive understanding of the relationship between host skin and skin microbiome.

In conclusion, our long-term study found that there was seasonal variation in skin microbial composition while maintaining an individual’s unique microbiome profile, and a significant association between the abundance of *Cutibacterium* and skin barrier parameter, TEWL. This study can contributed to a better comprehension of the skin microbiome and its intricate interplay with skin characteristics. Further functional studies on the changes in microbiome composition and skin barrier functions are needed for a deeper understanding of the skin microbiome’s contributions to skin biophysical characteristics.

## Data availability statement

The datasets presented in this study are deposited in the NCBI repository under accession number PRJNA1034158.

## Ethics statement

The studies involving humans were approved by the institutional review board at the LG H&H Research Center. The studies were conducted in accordance with the local legislation and institutional requirements. The participants provided their written informed consent to participate in this study.

## Author contributions

JS: Conceptualization, Data curation, Investigation, Methodology, Validation, Visualization, Writing – original draft, Writing – review & editing. SY: Conceptualization, Data curation, Formal analysis, Investigation, Methodology, Validation, Visualization, Writing – original draft, Writing – review & editing. K-NG: Conceptualization, Data curation, Formal analysis, Investigation, Methodology, Validation, Visualization, Writing – original draft, Writing – review & editing. HK: Conceptualization, Investigation, Validation, Visualization, Writing – review & editing. J-GS: Conceptualization, Investigation, Methodology, Validation, Writing – original draft, Writing – review & editing. SL: Conceptualization, Formal analysis, Investigation, Methodology, Validation, Writing – original draft, Writing – review & editing. BH: Conceptualization, Methodology, Validation, Writing – review & editing. YK: Conceptualization, Funding acquisition, Project administration, Resources, Supervision, Writing – review & editing. NK: Conceptualization, Funding acquisition, Project administration, Resources, Supervision, Writing – review & editing.
