# Supplementary material for: Longitudinal study of the interplay between the skin barrier and facial microbiome over 1 year
Source: Front Microbiol. 2023 Nov 16;14:1298632. doi: 10.3389/fmicb.2023.1298632 (PMC10687563; doi:10.3389/fmicb.2023.1298632)
Supplement: Supplementary file 1 [file Data_Sheet_1.pdf]

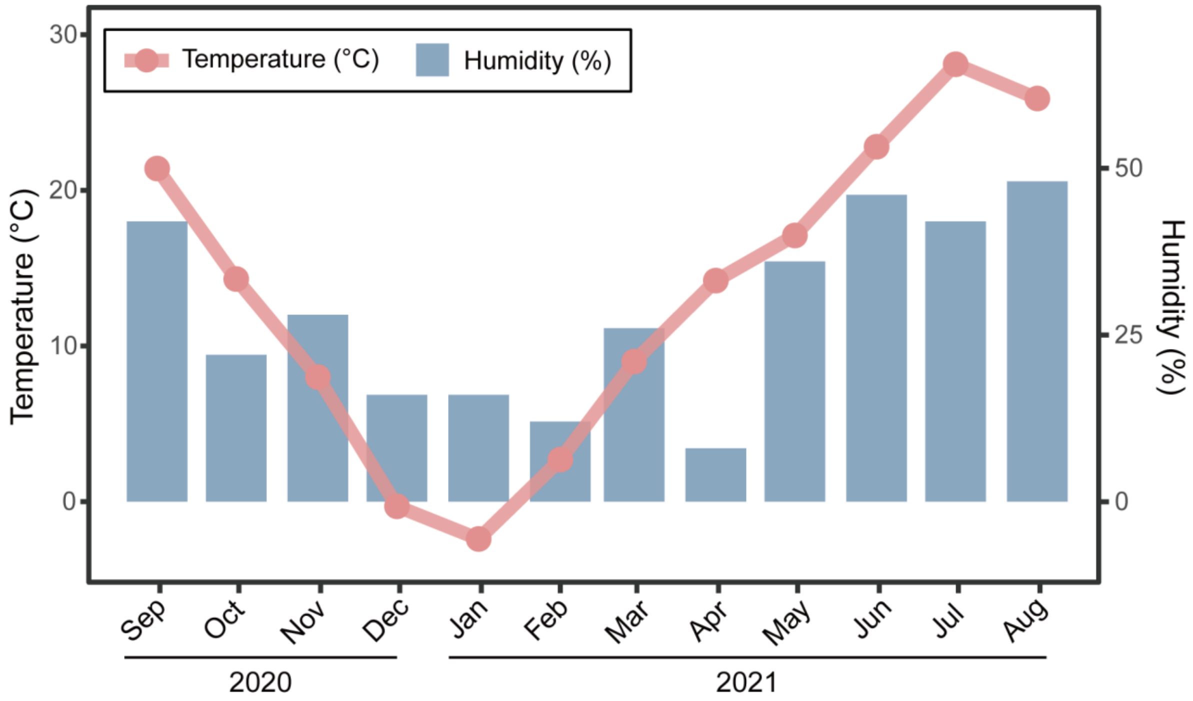

Supplementary Figure S1. Monthly variations of temperature and humidity. The line graph and dots represents the monthly mean temperature values, while the bar plot represents monthly humidity of Seoul, South Korea. These data were downloaded from the Korea Meteorological Administration.

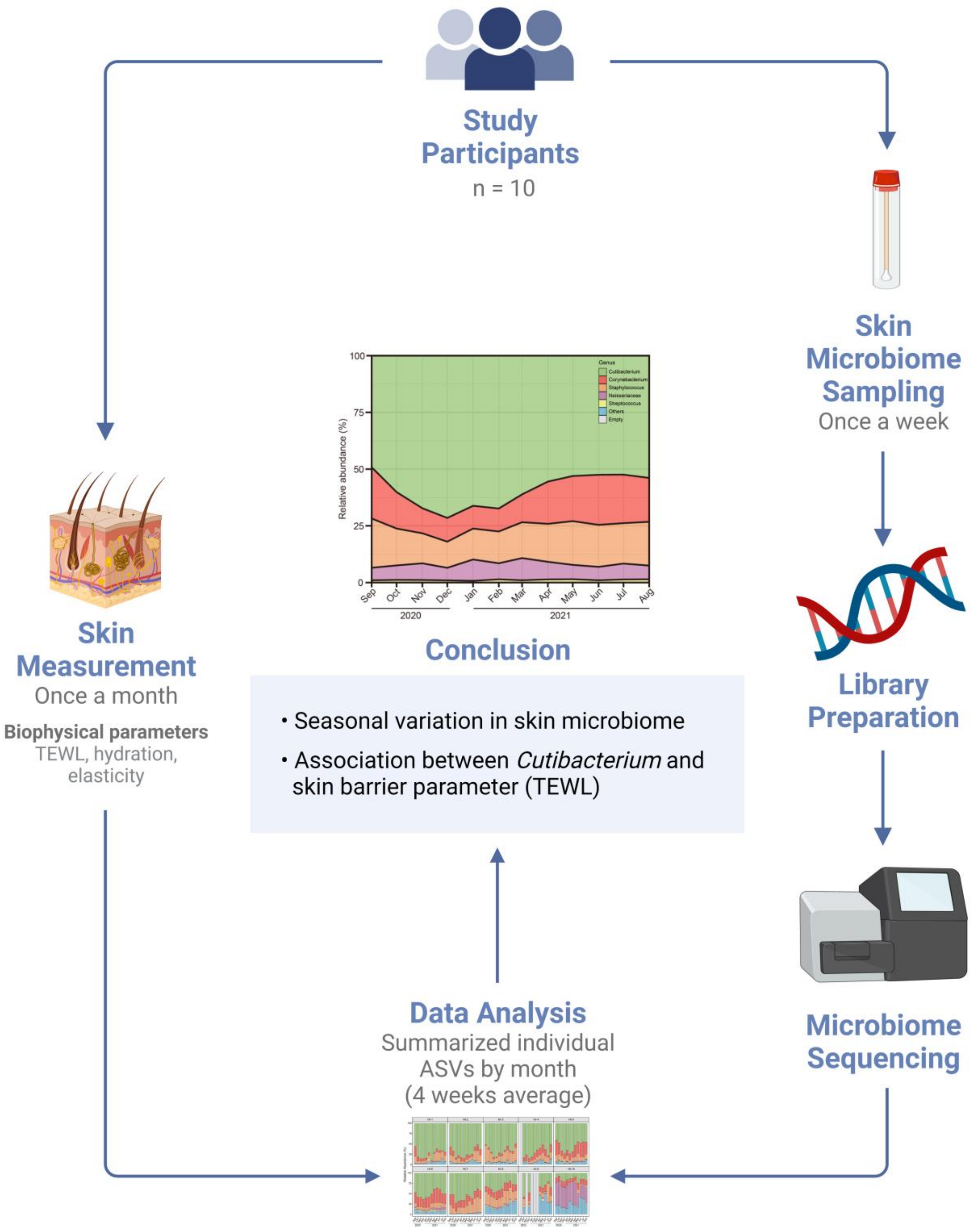

Supplementary Figure S2. Overview of study workflow

ASV, amplicon sequence variant.

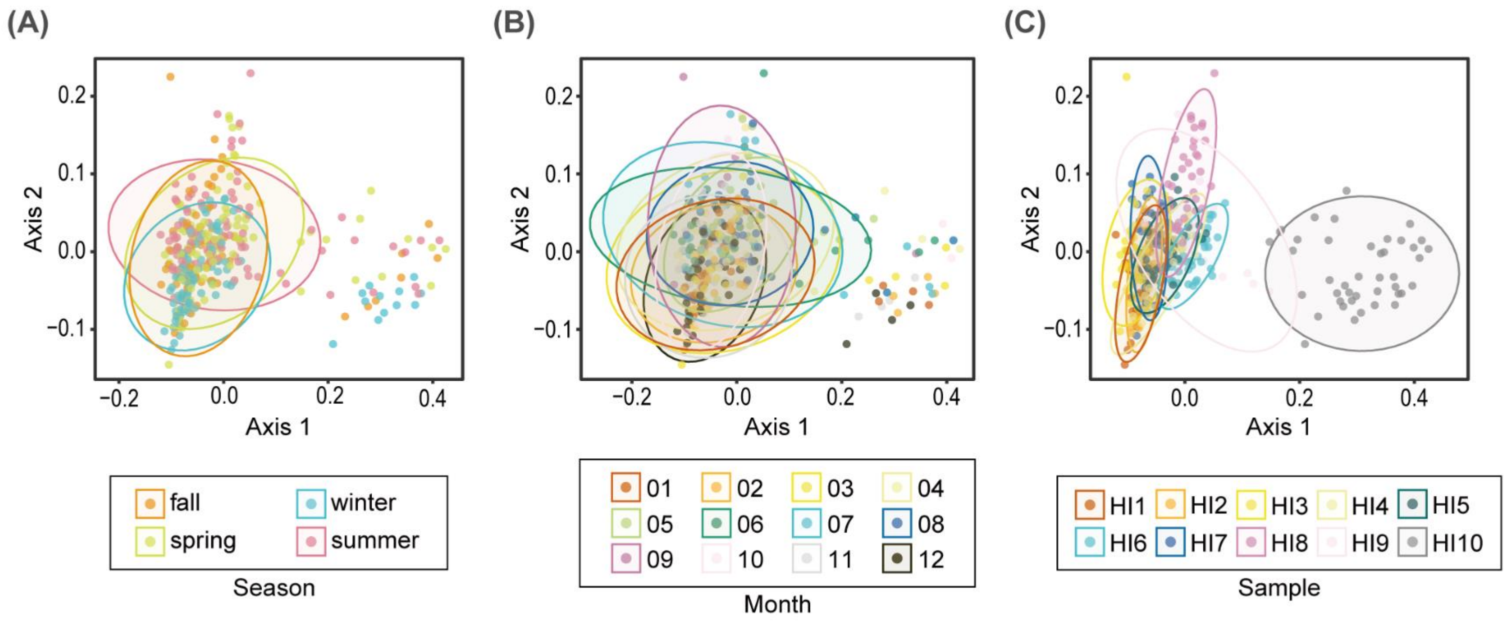

Supplementary Figure S3. β-diversity plot based on Jensen-Shannon distance. The multidimensional scaling plots of β-diversity according to (a) season, (b) month, and (c) study participants. Ellipses represents the 95% confidence area. HI, healthy individual.

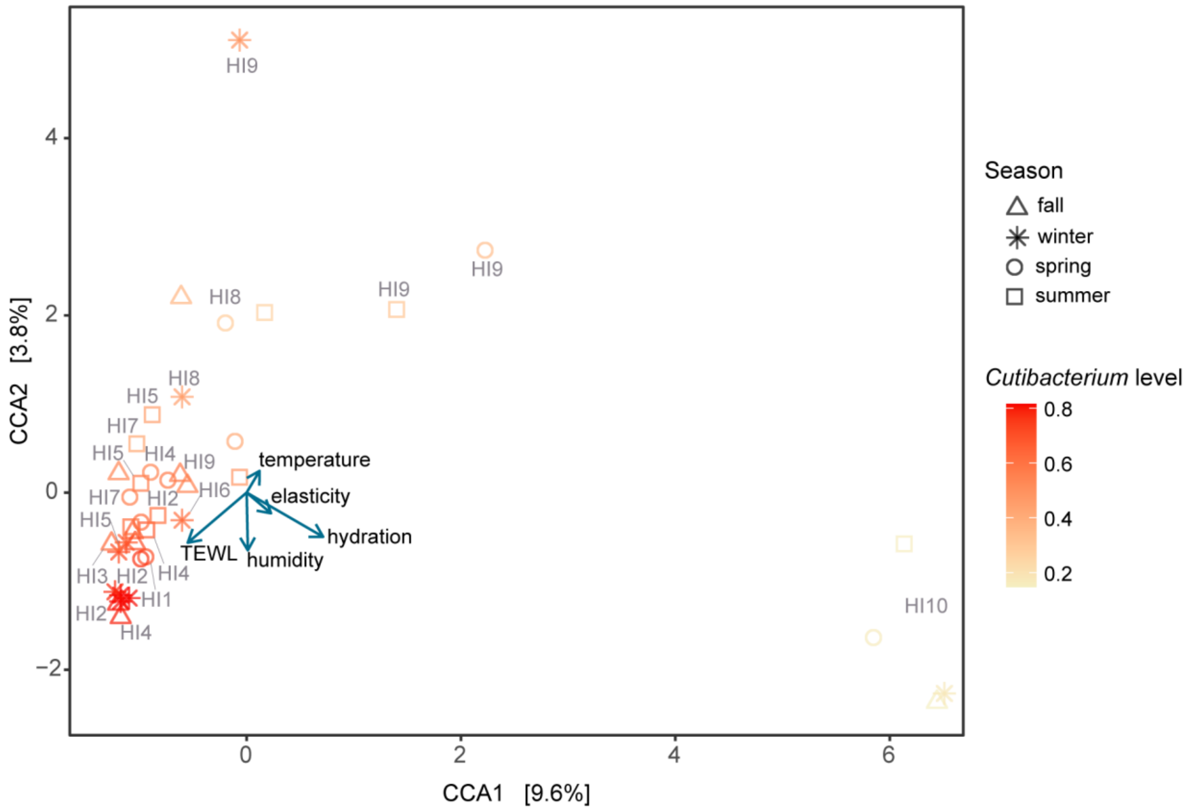

Supplementary Figure S4. Canonical correspondence analysis (CCA) plot of skin microbiome. Distribution of each participant's microbiome labeled by: open-triangles: fall, snow-flakes: winter, open-circles: spring, open-squares: summer. The relative abundance of *Cutibacterium* was represented by a red-colored gradation. The length of each arrow reflects the strength of the variable in explaining the observed dispersion of the microbiome. HI, healthy individual; CCA, canonical correspondence analysis.

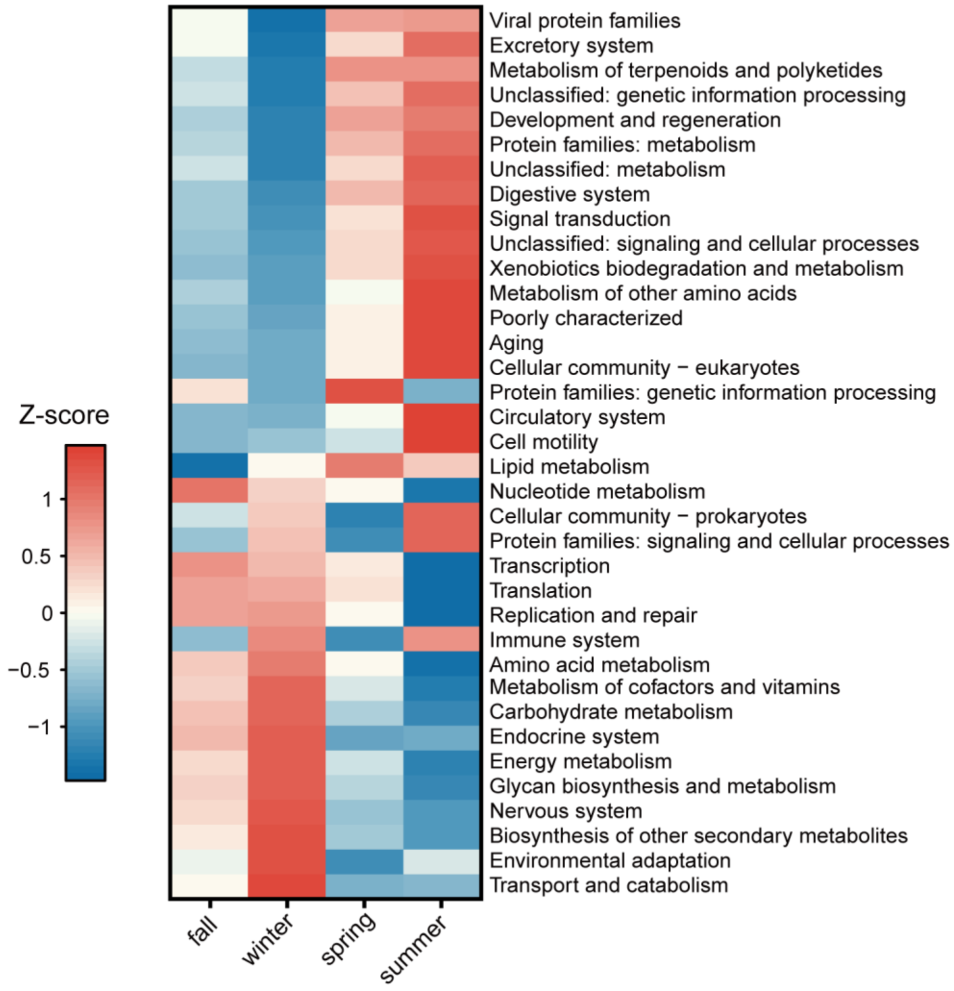

Supplementary Figure S5. Functional enrichment of skin microbiome according to the season. A heatmap of 36 pathways with significantly different enriched pathways inferred by PICRUSt2 according to the season. The degree of enrichment of each pathway was standardized to Z-score. The green color indicates lower abundance and the red color has a higher abundance.

Supplementary Table S1. Summary of study participants' skin biophysical parameters

Periods	Skin biophysical parameters		
	TEWL (Mean ± SD)	Hydration (Mean ± SD)	Elasticity (R5) (Mean ± SD)
2020 Fall	23.27 ± 4.47	64.93 ± 10.04	0.77 ± 0.06
2021 Winter	22.33 ± 7.03	53.87 ± 21.50	0.81 ± 0.05
2021 Spring	19.57 ± 5.36	62.36 ± 18.33	0.86 ± 0.04
2021 Summer	15.08 ± 3.41	63.52 ± 12.32	0.93 ± 0.04

Abbreviations: SD, standard deviation; TEWL, transepidermal water loss.

Supplementary Table S2. Pairwise PERMANOVA results of all taxa grouped by season

	Season	P-value			
		2020 Fall	2021 Winter	2021 Spring	2021 Summer
F-statistics[a]	2020 Fall	-	0.09	**0.03**	**0.005**
	2021 Winter	2.132	-	**0.001**	**0.001**
	2021 Spring	2.720	8.763	-	**0.04**
	2021 Summer	4.292	13.22	2.389	**-**

The permuted P-value of PERMANOVA pairwise comparisons of all taxa grouped by season.
P-values are based on 999 permutations and significant are shown in bold face (P-value < 0.05).
Abbreviation: PERMANOVA, permutational multivariate analysis of variance.
a, Pseudo F statistic for testing the null hypothesis of no differences.

Supplementary Table S3. Pairwise PERMANOVA results of all taxa grouped by month

	Month	P-value											
		Sep-20	Oct-20	Nov-20	Dec-20	Jan-21	Feb-21	Mar-21	Apr-21	May-21	Jun-21	Jul-21	Aug-21
F-statistics[a]	Sep-20	-	0.56	**0.03**	**0.006**	**0.03**	**0.04**	0.22	0.56	0.32	0.59	0.51	0.43
	Oct-20	0.804	-	0.42	0.08	0.40	0.47	0.75	0.53	0.06	0.25	0.28	0.15
	Nov-20	2.825	0.961	-	0.79	0.80	0.78	0.47	**0.02**	**0.001**	**0.01**	**0.01**	**0.002**
	Dec-20	4.867	2.120	0.408	-	0.39	0.34	0.10	**0.004**	**0.001**	**0.002**	**0.002**	**0.001**
	Jan-21	2.635	0.949	0.391	0.898	-	0.97	0.78	0.07	**0.002**	**0.02**	**0.02**	**0.004**
	Feb-21	2.601	0.873	0.447	1.029	0.219	-	0.78	0.07	**0.002**	**0.02**	**0.02**	**0.003**
	Mar-21	1.447	0.492	0.835	2.044	0.399	0.404	-	0.33	**0.04**	0.15	0.15	0.07
	Apr-21	0.767	0.796	3.068	5.514	2.376	2.321	1.044	-	0.48	0.50	0.42	0.16
	May-21	1.094	2.053	6.209	10.48	4.995	5.034	2.579	0.845	-	0.15	0.11	0.12
	Jun-21	0.710	1.258	3.821	6.775	3.308	3.235	1.638	0.844	1.576	-	0.90	0.31
	Jul-21	0.798	1.214	3.612	6.449	3.174	3.232	1.665	0.957	1.836	0.382	-	0.46
	Aug-21	0.943	1.542	4.957	9.014	4.324	4.332	2.293	1.459	1.670	1.114	0.893	-

The permuted P-value of PERMANOVA pairwise comparisons of all taxa grouped by month.
P-values are based on 999 permutations and significant are shown in bold face (P-value < 0.05).
Abbreviation: PERMANOVA, permutational multivariate analysis of variance.
a, Pseudo F statistic for testing the null hypothesis of no differences.

Supplementary Table S4. Pairwise PERMANOVA results of all taxa grouped by study participant

Study participant ID		P-value									
		HI-1	HI-2	HI-3	HI-4	HI-5	HI-6	HI-7	HI-8	HI-9	HI-10
F-statistics[a]	HI-1	**-**	**0.001**	**0.001**	**0.001**	**0.001**	**0.001**	**0.001**	**0.001**	**0.001**	**0.001**
	HI-2	5.967	-	**0.001**	**0.002**	**0.001**	**0.001**	**0.001**	**0.001**	**0.001**	**0.001**
	HI-3	12.47	16.19	-	**0.001**	**0.001**	**0.001**	**0.001**	**0.001**	**0.03**	**0.001**
	HI-4	7.815	6.954	15.68	-	**0.02**	**0.005**	**0.02**	**0.001**	**0.02**	**0.001**
	HI-5	33.79	24.77	21.60	3.427	-	**0.001**	**0.001**	**0.001**	**0.001**	**0.001**
	HI-6	62.17	59.72	70.34	4.867	19.24	-	**0.001**	**0.001**	**0.006**	**0.001**
	HI-7	27.49	25.50	16.55	3.498	9.652	30.16	-	**0.001**	**0.001**	**0.001**
	HI-8	76.04	74.28	56.30	41.09	46.09	48.72	43.78	-	**0.001**	**0.001**
	HI-9	23.65	22.16	2.942	2.855	4.698	3.471	20.01	5.488	-	**0.004**
	HI-10	231.8	198.7	162.4	36.35	155.4	143.1	210.8	152.0	5.865	**-**

The permuted P-value of PERMANOVA pairwise comparisons of all taxa grouped by study participant.
P-values are based on 999 permutations and significant are shown in bold face (P-value < 0.05).
Abbreviations: PERMANOVA, permutational multivariate analysis of variance; HI, healthy individual.
a, Pseudo F statistic for testing the null hypothesis of no differences.

Supplementary Table S5. Summer and winter KEGG path comparison results at the second level

Pathway	Summer-winter	
	Wilcoxon rank sum test P-value	FDR P-value
Unclassified: metabolism	**4.50E-23**	**1.80E-21**
Biosynthesis of other secondary metabolites	**2.31E-20**	**4.61E-19**
Digestive system	**1.89E-19**	**2.52E-18**
Carbohydrate metabolism	**7.68E-18**	**7.15E-17**
Protein families: metabolism	**8.94E-18**	**7.15E-17**
Energy metabolism	**3.19E-17**	**2.13E-16**
Unclassified: genetic information processing	**2.74E-16**	**1.57E-15**
Metabolism of cofactors and vitamins	**4.06E-16**	**2.03E-15**
Signal transduction	**1.36E-15**	**6.04E-15**
Excretory system	**4.28E-15**	**1.71E-14**
Xenobiotics biodegradation and metabolism	**6.89E-15**	**2.50E-14**
Poorly characterized	**8.89E-14**	**2.96E-13**
Unclassified: signaling and cellular processes	**1.30E-13**	**3.99E-13**
Metabolism of other amino acids	**4.78E-13**	**1.37E-12**
Development and regeneration	**7.85E-13**	**2.09E-12**
Viral protein families	**4.36E-12**	**1.09E-11**
Aging	**4.01E-11**	**9.44E-11**
Transport and catabolism	**9.30E-10**	**2.07E-09**
Circulatory system	**8.37E-09**	**1.76E-08**
Immune system	**2.50E-08**	**5.00E-08**
Metabolism of terpenoids and polyketides	**4.48E-08**	**8.54E-08**
Nervous system	**6.28E-08**	**1.14E-07**
Environmental adaptation	**5.29E-07**	**8.93E-07**
Replication and repair	**5.36E-07**	**8.93E-07**
Endocrine system	**1.11E-05**	**1.77E-05**
Protein families: genetic information processing	**2.02E-05**	**3.11E-05**
Translation	**5.69E-05**	**8.43E-05**
Cell motility	**2.05E-04**	**2.93E-04**
Amino acid metabolism	**6.04E-04**	**8.33E-04**
Cellular community - eukaryotes	**8.49E-04**	**1.13E-03**
Glycan biosynthesis and metabolism	**2.11E-03**	**2.72E-03**
Transcription	**8.48E-03**	**1.06E-02**
Cellular community - prokaryotes	**3.24E-02**	**3.93E-02**
Protein families: signaling and cellular processes	1.27E-01	1.49E-01
Sensory system	1.78E-01	2.03E-01
Nucleotide metabolism	3.13E-01	3.48E-01
Membrane transport	3.54E-01	3.83E-01
Lipid metabolism	4.72E-01	4.97E-01
Signaling molecules and interaction	5.32E-01	5.45E-01
Cell growth and death	6.28E-01	6.28E-01

Significant differences are shown in bold face (P-value < 0.05).
Abbreviations: KEGG, Kyoto Encyclopedia of Genes and Genomes; FDR, false discovery rate.

Supplementary Table S6. Summer and winter KEGG path comparison results at the third level

Pathway	Summer-winter	
	Wilcoxon rank sum test P-value	FDR P-value
Taurine and hypotaurine metabolism [PATH:ko00430]	**3.17E-22**	**1.22E-19**
Fat digestion and absorption [PATH:ko04975]	**1.33E-21**	**2.57E-19**
Nitrogen metabolism [PATH:ko00910]	**3.16E-21**	**4.06E-19**
Glycosphingolipid biosynthesis - globo and isoglobo series [PATH:ko00603]	**9.44E-21**	**9.09E-19**
MAPK signaling pathway - plant [PATH:ko04016]	**6.42E-20**	**3.78E-18**
Vitamin B6 metabolism [PATH:ko00750]	**6.88E-20**	**3.78E-18**
Glycerophospholipid metabolism [PATH:ko00564]	**5.33E-20**	**3.78E-18**
Citrate cycle (TCA cycle) [PATH:ko00020]	**1.07E-19**	**5.13E-18**
Glycosaminoglycan degradation [PATH:ko00531]	**1.69E-19**	**6.77E-18**
Xylene degradation [PATH:ko00622]	**1.93E-19**	**6.77E-18**
Unclassified viral proteins	**1.76E-19**	**6.77E-18**
Transcription	**2.54E-19**	**7.31E-18**
Dioxin degradation [PATH:ko00621]	**2.66E-19**	**7.31E-18**
Glycosphingolipid biosynthesis - ganglio series [PATH:ko00604]	**2.85E-19**	**7.31E-18**
Various types of N-glycan biosynthesis [PATH:ko00513]	**2.85E-19**	**7.31E-18**
Secondary bile acid biosynthesis [PATH:ko00121]	**4.69E-19**	**1.11E-17**
Oxidative phosphorylation [PATH:ko00190]	**4.90E-19**	**1.11E-17**
RIG-I-like receptor signaling pathway [PATH:ko04622]	**6.42E-19**	**1.37E-17**
Pentose phosphate pathway [PATH:ko00030]	**8.41E-19**	**1.70E-17**
Phenylpropanoid biosynthesis [PATH:ko00940]	**9.20E-19**	**1.77E-17**
Penicillin and cephalosporin biosynthesis [PATH:ko00311]	**1.18E-18**	**2.06E-17**
Nucleotide metabolism	**1.18E-18**	**2.06E-17**
Enzymes with EC numbers	**1.26E-18**	**2.11E-17**
Proteasome [PATH:ko03050]	**1.38E-18**	**2.21E-17**
Inositol phosphate metabolism [PATH:ko00562]	**1.61E-18**	**2.48E-17**
FoxO signaling pathway [PATH:ko04068]	**1.76E-18**	**2.60E-17**
Pyrimidine metabolism [PATH:ko00240]	**2.99E-18**	**4.26E-17**
Ether lipid metabolism [PATH:ko00565]	**3.26E-18**	**4.49E-17**
Primary bile acid biosynthesis [PATH:ko00120]	**7.68E-18**	**1.02E-16**
Dopaminergic synapse [PATH:ko04728]	**9.97E-18**	**1.24E-16**
Serotonergic synapse [PATH:ko04726]	**9.97E-18**	**1.24E-16**
Translation	**1.09E-17**	**1.31E-16**
Glycolysis / Gluconeogenesis [PATH:ko00010]	**1.14E-17**	**1.32E-16**
Base excision repair [PATH:ko03410]	**1.29E-17**	**1.46E-16**
Fructose and mannose metabolism [PATH:ko00051]	**1.35E-17**	**1.49E-16**
Fatty acid degradation [PATH:ko00071]	**1.91E-17**	**2.04E-16**
Streptomycin biosynthesis [PATH:ko00521]	**2.27E-17**	**2.36E-16**
Chlorocyclohexane and chlorobenzene degradation [PATH:ko00361]	**2.58E-17**	**2.61E-16**
Carbohydrate metabolism	**3.06E-17**	**3.02E-16**
Benzoate degradation [PATH:ko00362]	**3.33E-17**	**3.21E-16**
Biofilm formation - Pseudomonas aeruginosa [PATH:ko02025]	**4.59E-17**	**4.31E-16**

Porphyrin and chlorophyll metabolism [PATH:ko00860]	**5.00E-17**	**4.58E-16**
Glucagon signaling pathway [PATH:ko04922]	**5.21E-17**	**4.67E-16**
Antimicrobial resistance genes [BR:ko01504]	**7.17E-17**	**6.27E-16**
Longevity regulating pathway - multiple species [PATH:ko04213]	**9.63E-17**	**8.24E-16**
Peptidases and inhibitors [BR:ko01002]	**1.96E-16**	**1.64E-15**
HIF-1 signaling pathway [PATH:ko04066]	**2.58E-16**	**2.11E-15**
Biofilm formation - Vibrio cholerae [PATH:ko05111]	**3.44E-16**	**2.76E-15**
Prodigiosin biosynthesis [PATH:ko00333]	**3.52E-16**	**2.76E-15**
Secretion system [BR:ko02044]	**3.90E-16**	**3.00E-15**
Galactose metabolism [PATH:ko00052]	**4.50E-16**	**3.40E-15**
Carbon fixation pathways in prokaryotes [PATH:ko00720]	**4.60E-16**	**3.40E-15**
Two-component system [PATH:ko02020]	**7.23E-16**	**5.25E-15**
MAPK signaling pathway - fly [PATH:ko04013]	**1.04E-15**	**7.44E-15**
Starch and sucrose metabolism [PATH:ko00500]	**1.57E-15**	**1.10E-14**
alpha-Linolenic acid metabolism [PATH:ko00592]	**1.84E-15**	**1.24E-14**
Autophagy - yeast [PATH:ko04138]	**1.84E-15**	**1.24E-14**
Membrane trafficking [BR:ko04131]	**1.88E-15**	**1.25E-14**
Other glycan degradation [PATH:ko00511]	**2.16E-15**	**1.41E-14**
Replication and repair	**2.30E-15**	**1.47E-14**
Ubiquinone and other terpenoid-quinone biosynthesis [PATH:ko00130]	**2.87E-15**	**1.81E-14**
Bacterial secretion system [PATH:ko03070]	**3.30E-15**	**2.05E-14**
Proximal tubule bicarbonate reclamation [PATH:ko04964]	**4.28E-15**	**2.61E-14**
Transcription machinery [BR:ko03021]	**6.24E-15**	**3.75E-14**
Ethylbenzene degradation [PATH:ko00642]	**7.91E-15**	**4.68E-14**
PI3K-Akt signaling pathway [PATH:ko04151]	**9.44E-15**	**5.51E-14**
Carotenoid biosynthesis [PATH:ko00906]	**9.63E-15**	**5.53E-14**
Sphingolipid metabolism [PATH:ko00600]	**9.82E-15**	**5.56E-14**
Pentose and glucuronate interconversions [PATH:ko00040]	**1.45E-14**	**8.00E-14**
Nicotinate and nicotinamide metabolism [PATH:ko00760]	**1.45E-14**	**8.00E-14**
Amino sugar and nucleotide sugar metabolism [PATH:ko00520]	**1.67E-14**	**9.04E-14**
Ascorbate and aldarate metabolism [PATH:ko00053]	**2.61E-14**	**1.39E-13**
Ferroptosis [PATH:ko04216]	**2.66E-14**	**1.40E-13**
RNA degradation [PATH:ko03018]	**2.76E-14**	**1.44E-13**
Drug metabolism - other enzymes [PATH:ko00983]	**4.39E-14**	**2.25E-13**
Monobactam biosynthesis [PATH:ko00261]	**5.21E-14**	**2.64E-13**
Phenazine biosynthesis [PATH:ko00405]	**5.85E-14**	**2.92E-13**
Exosome [BR:ko04147]	**6.08E-14**	**3.00E-13**
General function prediction only	**6.31E-14**	**3.08E-13**
Ribosome biogenesis in eukaryotes [PATH:ko03008]	**8.72E-14**	**4.20E-13**
Two-component system [BR:ko02022]	**8.89E-14**	**4.23E-13**
Thermogenesis [PATH:ko04714]	**9.77E-14**	**4.59E-13**
Novobiocin biosynthesis [PATH:ko00401]	**1.05E-13**	**4.89E-13**
Mineral absorption [PATH:ko04978]	**1.18E-13**	**5.41E-13**
Carbapenem biosynthesis [PATH:ko00332]	**1.32E-13**	**5.99E-13**

Transporters [BR:ko02000]	**1.54E-13**	**6.88E-13**
Cyanoamino acid metabolism [PATH:ko00460]	**1.69E-13**	**7.47E-13**
Biosynthesis of siderophore group nonribosomal peptides [PATH:ko01053]	**1.82E-13**	**7.96E-13**
Cofactor metabolism	**2.24E-13**	**9.67E-13**
Quorum sensing [PATH:ko02024]	**2.45E-13**	**1.04E-12**
Transport	**2.45E-13**	**1.04E-12**
Histidine metabolism [PATH:ko00340]	**2.50E-13**	**1.05E-12**
Prolactin signaling pathway [PATH:ko04917]	**2.74E-13**	**1.14E-12**
Nucleotide excision repair [PATH:ko03420]	**2.96E-13**	**1.21E-12**
Phospholipase D signaling pathway [PATH:ko04072]	**4.87E-13**	**1.97E-12**
Axon regeneration [PATH:ko04361]	**5.80E-13**	**2.33E-12**
Lysosome [PATH:ko04142]	**6.66E-13**	**2.64E-12**
Basal transcription factors [PATH:ko03022]	**7.03E-13**	**2.76E-12**
Acarbose and validamycin biosynthesis [PATH:ko00525]	**7.99E-13**	**3.11E-12**
Atrazine degradation [PATH:ko00791]	**1.74E-12**	**6.70E-12**
Chromosome and associated proteins [BR:ko03036]	**2.01E-12**	**7.59E-12**
Sulfur metabolism [PATH:ko00920]	**2.01E-12**	**7.59E-12**
Longevity regulating pathway [PATH:ko04211]	**2.45E-12**	**9.15E-12**
Plant-pathogen interaction [PATH:ko04626]	**2.98E-12**	**1.10E-11**
Meiosis - yeast [PATH:ko04113]	**3.96E-12**	**1.45E-11**
Lipid metabolism	**3.99E-12**	**1.45E-11**
Viral proteins [BR:ko03200]	**4.36E-12**	**1.57E-11**
Bacterial toxins [BR:ko02042]	**4.64E-12**	**1.65E-11**
Chloroalkane and chloroalkene degradation [PATH:ko00625]	**5.07E-12**	**1.79E-11**
Endocytosis [PATH:ko04144]	**8.42E-12**	**2.95E-11**
Biosynthesis of vancomycin group antibiotics [PATH:ko01055]	**1.17E-11**	**4.06E-11**
Energy metabolism	**1.21E-11**	**4.13E-11**
Toll and Imd signaling pathway [PATH:ko04624]	**1.20E-11**	**4.13E-11**
Messenger RNA biogenesis [BR:ko03019]	**1.44E-11**	**4.87E-11**
Protein processing	**1.68E-11**	**5.63E-11**
N-Glycan biosynthesis [PATH:ko00510]	**2.72E-11**	**9.02E-11**
Phosphatidylinositol signaling system [PATH:ko04070]	**3.50E-11**	**1.13E-10**
Mitochondrial biogenesis [BR:ko03029]	**3.45E-11**	**1.13E-10**
Propanoate metabolism [PATH:ko00640]	**3.50E-11**	**1.13E-10**
Methane metabolism [PATH:ko00680]	**3.88E-11**	**1.24E-10**
Peptidoglycan biosynthesis and degradation proteins [BR:ko01011]	**4.08E-11**	**1.30E-10**
Metabolism of xenobiotics by cytochrome P450 [PATH:ko00980]	**4.99E-11**	**1.58E-10**
Peptidoglycan biosynthesis [PATH:ko00550]	**6.64E-11**	**2.08E-10**
Tyrosine metabolism [PATH:ko00350]	**7.22E-11**	**2.24E-10**
Glycan metabolism	**9.11E-11**	**2.80E-10**
Biosynthesis of various secondary metabolites - part 3 [PATH:ko00997]	**1.37E-10**	**4.20E-10**
Adipocytokine signaling pathway [PATH:ko04920]	**2.04E-10**	**6.13E-10**
Signaling proteins	**2.04E-10**	**6.13E-10**
Cell motility	**2.17E-10**	**6.49E-10**

Function unknown	**2.44E-10**	**7.21E-10**
Arginine and proline metabolism [PATH:ko00330]	**2.73E-10**	**8.02E-10**
D-Amino acid metabolism [PATH:ko00470]	**4.94E-10**	**1.44E-09**
Lipopolysaccharide biosynthesis proteins [BR:ko01005]	**5.35E-10**	**1.55E-09**
Non-homologous end-joining [PATH:ko03450]	**5.70E-10**	**1.63E-09**
Thyroid hormone synthesis [PATH:ko04918]	**5.70E-10**	**1.63E-09**
Signaling pathways regulating pluripotency of stem cells [PATH:ko04550]	**6.74E-10**	**1.91E-09**
One carbon pool by folate [PATH:ko00670]	**6.90E-10**	**1.94E-09**
Proteasome [BR:ko03051]	**8.20E-10**	**2.29E-09**
beta-Alanine metabolism [PATH:ko00410]	**9.90E-10**	**2.74E-09**
Naphthalene degradation [PATH:ko00626]	**1.14E-09**	**3.11E-09**
Glycosaminoglycan binding proteins [BR:ko00536]	**1.14E-09**	**3.11E-09**
Secondary metabolism	**1.21E-09**	**3.29E-09**
Antigen processing and presentation [PATH:ko04612]	**1.83E-09**	**4.83E-09**
Estrogen signaling pathway [PATH:ko04915]	**1.83E-09**	**4.83E-09**
Progesterone-mediated oocyte maturation [PATH:ko04914]	**1.83E-09**	**4.83E-09**
Th17 cell differentiation [PATH:ko04659]	**1.83E-09**	**4.83E-09**
Pantothenate and CoA biosynthesis [PATH:ko00770]	**1.90E-09**	**4.98E-09**
IL-17 signaling pathway [PATH:ko04657]	**2.10E-09**	**5.47E-09**
Protein phosphatases and associated proteins [BR:ko01009]	**2.19E-09**	**5.65E-09**
Homologous recombination [PATH:ko03440]	**2.40E-09**	**6.15E-09**
Prokaryotic defense system [BR:ko02048]	**2.47E-09**	**6.30E-09**
NOD-like receptor signaling pathway [PATH:ko04621]	**4.47E-09**	**1.13E-08**
Glycosyltransferases [BR:ko01003]	**4.96E-09**	**1.25E-08**
Steroid degradation [PATH:ko00984]	**5.35E-09**	**1.34E-08**
Lysine degradation [PATH:ko00310]	**6.22E-09**	**1.54E-08**
Apoptosis [PATH:ko04210]	**7.89E-09**	**1.95E-08**
Cardiac muscle contraction [PATH:ko04260]	**8.13E-09**	**1.99E-08**
Amino acid related enzymes [BR:ko01007]	**8.25E-09**	**2.01E-08**
Transfer RNA biogenesis [BR:ko03016]	**8.37E-09**	**2.03E-08**
Pancreatic secretion [PATH:ko04972]	**9.22E-09**	**2.22E-08**
Styrene degradation [PATH:ko00643]	**9.28E-09**	**2.22E-08**
Salivary secretion [PATH:ko04970]	**9.63E-09**	**2.29E-08**
Apoptosis - fly [PATH:ko04214]	**1.11E-08**	**2.62E-08**
Nitrotoluene degradation [PATH:ko00633]	**1.16E-08**	**2.72E-08**
Ribosome biogenesis [BR:ko03009]	**1.18E-08**	**2.74E-08**
Carbohydrate digestion and absorption [PATH:ko04973]	**1.30E-08**	**3.02E-08**
Fluorobenzoate degradation [PATH:ko00364]	**1.42E-08**	**3.28E-08**
Cell cycle - Caulobacter [PATH:ko04112]	**1.62E-08**	**3.71E-08**
Linoleic acid metabolism [PATH:ko00591]	**1.93E-08**	**4.39E-08**
Isoquinoline alkaloid biosynthesis [PATH:ko00950]	**2.10E-08**	**4.76E-08**
Amino acid metabolism	**2.29E-08**	**5.16E-08**
Lipoic acid metabolism [PATH:ko00785]	**2.43E-08**	**5.44E-08**
Arabinogalactan biosynthesis - Mycobacterium [PATH:ko00572]	**2.54E-08**	**5.64E-08**

Polyketide biosynthesis proteins [BR:ko01008]	**2.65E-08**	**5.86E-08**
Betalain biosynthesis [PATH:ko00965]	**2.72E-08**	**5.99E-08**
Apoptosis - multiple species [PATH:ko04215]	**2.93E-08**	**6.37E-08**
p53 signaling pathway [PATH:ko04115]	**2.93E-08**	**6.37E-08**
Geraniol degradation [PATH:ko00281]	**4.48E-08**	**9.69E-08**
Phosphonate and phosphinate metabolism [PATH:ko00440]	**4.68E-08**	**1.01E-07**
Renin-angiotensin system [PATH:ko04614]	**4.74E-08**	**1.01E-07**
Sulfur relay system [PATH:ko04122]	**6.19E-08**	**1.32E-07**
Mismatch repair [PATH:ko03430]	**6.92E-08**	**1.46E-07**
CD molecules [BR:ko04090]	**7.07E-08**	**1.49E-07**
Steroid hormone biosynthesis [PATH:ko00140]	**7.19E-08**	**1.50E-07**
Flagellar assembly [PATH:ko02040]	**7.95E-08**	**1.66E-07**
Protein digestion and absorption [PATH:ko04974]	**1.24E-07**	**2.58E-07**
Protein processing in endoplasmic reticulum [PATH:ko04141]	**1.29E-07**	**2.65E-07**
Transcription factors [BR:ko03000]	**3.57E-07**	**7.31E-07**
Renin secretion [PATH:ko04924]	**3.99E-07**	**8.13E-07**
Nonribosomal peptide structures [PATH:ko01054]	**4.02E-07**	**8.14E-07**
Toluene degradation [PATH:ko00623]	**4.07E-07**	**8.21E-07**
Aminoacyl-tRNA biosynthesis [PATH:ko00970]	**4.70E-07**	**9.43E-07**
Translation factors [BR:ko03012]	**4.76E-07**	**9.50E-07**
Polycyclic aromatic hydrocarbon degradation [PATH:ko00624]	**6.18E-07**	**1.23E-06**
Bacterial motility proteins [BR:ko02035]	**7.03E-07**	**1.39E-06**
Pyruvate metabolism [PATH:ko00620]	**9.44E-07**	**1.85E-06**
Glyoxylate and dicarboxylate metabolism [PATH:ko00630]	**9.93E-07**	**1.94E-06**
Terpenoid backbone biosynthesis [PATH:ko00900]	**1.01E-06**	**1.96E-06**
Calcium signaling pathway [PATH:ko04020]	**1.05E-06**	**2.04E-06**
Tryptophan metabolism [PATH:ko00380]	**1.09E-06**	**2.09E-06**
Lipopolysaccharide biosynthesis [PATH:ko00540]	**1.10E-06**	**2.11E-06**
Staurosporine biosynthesis [PATH:ko00404]	**1.74E-06**	**3.31E-06**
RNA polymerase [PATH:ko03020]	**2.56E-06**	**4.86E-06**
cAMP signaling pathway [PATH:ko04024]	**2.58E-06**	**4.87E-06**
Necroptosis [PATH:ko04217]	**2.90E-06**	**5.44E-06**
O-Antigen repeat unit biosynthesis [PATH:ko00542]	**3.74E-06**	**6.99E-06**
Thyroid hormone signaling pathway [PATH:ko04919]	**4.22E-06**	**7.84E-06**
Arachidonic acid metabolism [PATH:ko00590]	**5.89E-06**	**1.09E-05**
DNA replication proteins [BR:ko03032]	**7.19E-06**	**1.33E-05**
Protein export [PATH:ko03060]	**8.77E-06**	**1.61E-05**
Sphingolipid signaling pathway [PATH:ko04071]	**9.74E-06**	**1.78E-05**
Biosynthesis of various secondary metabolites - part 2 [PATH:ko00998]	**1.39E-05**	**2.53E-05**
Purine metabolism [PATH:ko00230]	**1.63E-05**	**2.95E-05**
Arginine biosynthesis [PATH:ko00220]	**1.83E-05**	**3.28E-05**
Polyketide sugar unit biosynthesis [PATH:ko00523]	**1.97E-05**	**3.54E-05**
Ribosome [PATH:ko03010]	**2.02E-05**	**3.60E-05**
Ribosome [BR:ko03011]	**2.04E-05**	**3.62E-05**

Bile secretion [PATH:ko04976]	**3.68E-05**	**6.49E-05**
Neuroactive ligand-receptor interaction [PATH:ko04080]	**4.64E-05**	**8.16E-05**
AMPK signaling pathway [PATH:ko04152]	**4.75E-05**	**8.31E-05**
Cysteine and methionine metabolism [PATH:ko00270]	**6.00E-05**	**1.05E-04**
Insect hormone biosynthesis [PATH:ko00981]	**6.20E-05**	**1.07E-04**
Flavonoid biosynthesis [PATH:ko00941]	**8.85E-05**	**1.53E-04**
Limonene and pinene degradation [PATH:ko00903]	**1.88E-04**	**3.23E-04**
Cytochrome P450 [BR:ko00199]	**2.29E-04**	**3.91E-04**
Glycerolipid metabolism [PATH:ko00561]	**2.35E-04**	**4.01E-04**
Retinol metabolism [PATH:ko00830]	**3.40E-04**	**5.77E-04**
Biosynthesis of ansamycins [PATH:ko01051]	**3.78E-04**	**6.38E-04**
Ion channels [BR:ko04040]	**4.87E-04**	**8.19E-04**
Glycosylphosphatidylinositol (GPI)-anchored proteins [BR:ko00537]	**5.35E-04**	**8.96E-04**
Caffeine metabolism [PATH:ko00232]	**5.91E-04**	**9.85E-04**
Ras signaling pathway [PATH:ko04014]	**8.28E-04**	**1.37E-03**
Riboflavin metabolism [PATH:ko00740]	**1.00E-03**	**1.66E-03**
GnRH signaling pathway [PATH:ko04912]	**1.33E-03**	**2.18E-03**
Retrograde endocannabinoid signaling [PATH:ko04723]	**1.44E-03**	**2.36E-03**
Cell growth	**2.29E-03**	**3.74E-03**
Aminobenzoate degradation [PATH:ko00627]	**2.35E-03**	**3.81E-03**
O-Antigen nucleotide sugar biosynthesis [PATH:ko00541]	**2.43E-03**	**3.93E-03**
Domain-containing proteins not elsewhere classified [BR:ko04990]	**2.67E-03**	**4.30E-03**
DNA replication [PATH:ko03030]	**2.86E-03**	**4.59E-03**
Carbon fixation in photosynthetic organisms [PATH:ko00710]	**3.10E-03**	**4.96E-03**
Steroid biosynthesis [PATH:ko00100]	**3.28E-03**	**5.22E-03**
Phenylalanine metabolism [PATH:ko00360]	**3.36E-03**	**5.33E-03**
Lysine biosynthesis [PATH:ko00300]	**3.73E-03**	**5.89E-03**
Chemokine signaling pathway [PATH:ko04062]	**3.76E-03**	**5.91E-03**
Peroxisome [PATH:ko04146]	**4.48E-03**	**7.01E-03**
Folate biosynthesis [PATH:ko00790]	**5.15E-03**	**8.00E-03**
Melanogenesis [PATH:ko04916]	**5.15E-03**	**8.00E-03**
Bisphenol degradation [PATH:ko00363]	**5.23E-03**	**8.06E-03**
Glutathione metabolism [PATH:ko00480]	**5.23E-03**	**8.06E-03**
Protein kinases [BR:ko01001]	**5.74E-03**	**8.81E-03**
Rap1 signaling pathway [PATH:ko04015]	**6.42E-03**	**9.80E-03**
Fatty acid biosynthesis [PATH:ko00061]	**6.94E-03**	**1.06E-02**
ABC transporters [PATH:ko02010]	**7.42E-03**	**1.13E-02**
Ubiquitin system [BR:ko04121]	**1.02E-02**	**1.54E-02**
Photosynthesis - antenna proteins [PATH:ko00196]	**1.11E-02**	**1.67E-02**
Prenyltransferases [BR:ko01006]	**1.16E-02**	**1.73E-02**
Hematopoietic cell lineage [PATH:ko04640]	**1.34E-02**	**2.00E-02**
Lipid biosynthesis proteins [BR:ko01004]	**1.41E-02**	**2.10E-02**
Selenocompound metabolism [PATH:ko00450]	**1.56E-02**	**2.32E-02**
Fc gamma R-mediated phagocytosis [PATH:ko04666]	**2.07E-02**	**3.06E-02**

Longevity regulating pathway - worm [PATH:ko04212]	**2.58E-02**	**3.79E-02**
Biotin metabolism [PATH:ko00780]	**2.77E-02**	**4.05E-02**
Wnt signaling pathway [PATH:ko04310]	**2.95E-02**	**4.31E-02**
Structural proteins	**4.04E-02**	5.87E-02
Biosynthesis of type II polyketide products [PATH:ko01057]	**4.31E-02**	6.24E-02
Caprolactam degradation [PATH:ko00930]	**4.50E-02**	6.49E-02
mRNA surveillance pathway [PATH:ko03015]	**4.79E-02**	6.89E-02
Tetracycline biosynthesis [PATH:ko00253]	5.69E-02	8.15E-02
Zeatin biosynthesis [PATH:ko00908]	5.95E-02	8.45E-02
Insulin signaling pathway [PATH:ko04910]	5.95E-02	8.45E-02
Notch signaling pathway [PATH:ko04330]	6.14E-02	8.69E-02
G protein-coupled receptors [BR:ko04030]	6.27E-02	8.81E-02
Nucleocytoplasmic transport [PATH:ko03013]	6.27E-02	8.81E-02
Cytoskeleton proteins [BR:ko04812]	7.00E-02	9.80E-02
Osteoclast differentiation [PATH:ko04380]	7.85E-02	1.10E-01
DNA repair and recombination proteins [BR:ko03400]	1.13E-01	1.57E-01
Drug metabolism - cytochrome P450 [PATH:ko00982]	1.18E-01	1.63E-01
Biofilm formation - Escherichia coli [PATH:ko02026]	1.43E-01	1.97E-01
Hippo signaling pathway [PATH:ko04390]	1.54E-01	2.12E-01
Bacterial chemotaxis [PATH:ko02030]	1.56E-01	2.13E-01
Phagosome [PATH:ko04145]	1.57E-01	2.15E-01
Fatty acid elongation [PATH:ko00062]	1.63E-01	2.21E-01
Adherens junction [PATH:ko04520]	1.64E-01	2.22E-01
Neutrophil extracellular trap formation [PATH:ko04613]	1.84E-01	2.49E-01
Isoflavonoid biosynthesis [PATH:ko00943]	1.95E-01	2.62E-01
B cell receptor signaling pathway [PATH:ko04662]	1.99E-01	2.63E-01
Natural killer cell mediated cytotoxicity [PATH:ko04650]	1.99E-01	2.63E-01
T cell receptor signaling pathway [PATH:ko04660]	1.99E-01	2.63E-01
Th1 and Th2 cell differentiation [PATH:ko04658]	1.99E-01	2.63E-01
Butanoate metabolism [PATH:ko00650]	1.99E-01	2.63E-01
Leukocyte transendothelial migration [PATH:ko04670]	2.03E-01	2.67E-01
Tight junction [PATH:ko04530]	2.13E-01	2.79E-01
Oocyte meiosis [PATH:ko04114]	2.13E-01	2.79E-01
PPAR signaling pathway [PATH:ko03320]	2.15E-01	2.81E-01
Hippo signaling pathway - fly [PATH:ko04391]	2.22E-01	2.89E-01
Others	2.29E-01	2.96E-01
Phototransduction - fly [PATH:ko04745]	2.52E-01	3.26E-01
Lipoarabinomannan (LAM) biosynthesis [PATH:ko00571]	2.59E-01	3.33E-01
Sesquiterpenoid and triterpenoid biosynthesis [PATH:ko00909]	2.66E-01	3.41E-01
Oxytocin signaling pathway [PATH:ko04921]	2.85E-01	3.65E-01
Flavone and flavonol biosynthesis [PATH:ko00944]	2.97E-01	3.79E-01
MAPK signaling pathway [PATH:ko04010]	3.15E-01	4.00E-01
GTP-binding proteins [BR:ko04031]	3.25E-01	4.10E-01
VEGF signaling pathway [PATH:ko04370]	3.24E-01	4.10E-01

Ubiquitin mediated proteolysis [PATH:ko04120]	3.43E-01	4.31E-01
Cellular senescence [PATH:ko04218]	3.54E-01	4.44E-01
Phosphotransferase system (PTS) [PATH:ko02060]	3.57E-01	4.46E-01
Cell adhesion molecules [PATH:ko04514]	3.72E-01	4.63E-01
Axon guidance [PATH:ko04360]	3.73E-01	4.64E-01
Long-term potentiation [PATH:ko04720]	3.97E-01	4.91E-01
Ovarian steroidogenesis [PATH:ko04913]	3.99E-01	4.92E-01
Cortisol synthesis and secretion [PATH:ko04927]	4.19E-01	5.06E-01
Hedgehog signaling pathway - fly [PATH:ko04341]	4.19E-01	5.06E-01
Hedgehog signaling pathway [PATH:ko04340]	4.19E-01	5.06E-01
Taste transduction [PATH:ko04742]	4.19E-01	5.06E-01
Platelet activation [PATH:ko04611]	4.13E-01	5.06E-01
Photosynthesis [PATH:ko00195]	4.18E-01	5.06E-01
Spliceosome [PATH:ko03040]	4.17E-01	5.06E-01
Mannose type O-glycan biosynthesis [PATH:ko00515]	4.25E-01	5.09E-01
Other types of O-glycan biosynthesis [PATH:ko00514]	4.25E-01	5.09E-01
Spliceosome [BR:ko03041]	4.26E-01	5.09E-01
Thiamine metabolism [PATH:ko00730]	4.29E-01	5.11E-01
Gastric acid secretion [PATH:ko04971]	4.30E-01	5.11E-01
Endocrine and other factor-regulated calcium reabsorption [PATH:ko04961]	4.65E-01	5.51E-01
C5-Branched dibasic acid metabolism [PATH:ko00660]	4.68E-01	5.52E-01
Glucosinolate biosynthesis [PATH:ko00966]	4.80E-01	5.65E-01
Biosynthesis of various secondary metabolites - part 1 [PATH:ko00999]	4.84E-01	5.68E-01
Fc epsilon RI signaling pathway [PATH:ko04664]	4.89E-01	5.72E-01
Lectins [BR:ko04091]	4.97E-01	5.80E-01
Dorso-ventral axis formation [PATH:ko04320]	5.18E-01	6.02E-01
C-type lectin receptor signaling pathway [PATH:ko04625]	5.29E-01	6.14E-01
Chaperones and folding catalysts [BR:ko03110]	5.39E-01	6.18E-01
Cell adhesion molecules [BR:ko04515]	5.37E-01	6.18E-01
Insulin secretion [PATH:ko04911]	5.38E-01	6.18E-01
Fanconi anemia pathway [PATH:ko03460]	5.34E-01	6.18E-01
Gap junction [PATH:ko04540]	5.41E-01	6.18E-01
Proteoglycans [BR:ko00535]	5.62E-01	6.40E-01
Biosynthesis of enediyne antibiotics [PATH:ko01059]	5.72E-01	6.41E-01
TNF signaling pathway [PATH:ko04668]	5.73E-01	6.41E-01
NF-kappa B signaling pathway [PATH:ko04064]	5.71E-01	6.41E-01
Apelin signaling pathway [PATH:ko04371]	5.66E-01	6.41E-01
Cell cycle - yeast [PATH:ko04111]	5.71E-01	6.41E-01
Cell cycle [PATH:ko04110]	5.71E-01	6.41E-01
Neurotrophin signaling pathway [PATH:ko04722]	5.97E-01	6.66E-01
Indole alkaloid biosynthesis [PATH:ko00901]	6.18E-01	6.87E-01
Autophagy - animal [PATH:ko04140]	6.19E-01	6.87E-01
Mitophagy - animal [PATH:ko04137]	6.28E-01	6.95E-01
Furfural degradation [PATH:ko00365]	6.31E-01	6.96E-01

Relaxin signaling pathway [PATH:ko04926]	6.38E-01	7.02E-01
Plant hormone signal transduction [PATH:ko04075]	6.41E-01	7.03E-01
Biosynthesis of type II polyketide backbone [PATH:ko01056]	6.51E-01	7.12E-01
Type I polyketide structures [PATH:ko01052]	6.55E-01	7.14E-01
Regulation of lipolysis in adipocytes [PATH:ko04923]	6.64E-01	7.23E-01
ErbB signaling pathway [PATH:ko04012]	6.68E-01	7.25E-01
Monoterpenoid biosynthesis [PATH:ko00902]	6.73E-01	7.28E-01
Cholesterol metabolism [PATH:ko04979]	6.81E-01	7.34E-01
Vasopressin-regulated water reabsorption [PATH:ko04962]	6.98E-01	7.51E-01
MAPK signaling pathway - yeast [PATH:ko04011]	7.31E-01	7.81E-01
GABAergic synapse [PATH:ko04727]	7.31E-01	7.81E-01
Complement and coagulation cascades [PATH:ko04610]	7.34E-01	7.81E-01
Vitamin digestion and absorption [PATH:ko04977]	7.34E-01	7.81E-01
Olfactory transduction [PATH:ko04740]	7.38E-01	7.83E-01
Cholinergic synapse [PATH:ko04725]	7.87E-01	8.28E-01
Glycosaminoglycan biosynthesis - keratan sulfate [PATH:ko00533]	7.89E-01	8.28E-01
Glycosphingolipid biosynthesis - lacto and neolacto series [PATH:ko00601]	7.89E-01	8.28E-01
Long-term depression [PATH:ko04730]	7.89E-01	8.28E-01
Regulation of actin cytoskeleton [PATH:ko04810]	7.96E-01	8.32E-01
Aldosterone-regulated sodium reabsorption [PATH:ko04960]	7.99E-01	8.32E-01
Glutamatergic synapse [PATH:ko04724]	7.98E-01	8.32E-01
Photosynthesis proteins [BR:ko00194]	8.25E-01	8.56E-01
Adrenergic signaling in cardiomyocytes [PATH:ko04261]	8.36E-01	8.65E-01
Glycosaminoglycan biosynthesis - chondroitin sulfate / dermatan sulfate [PATH:ko00532]	8.69E-01	8.97E-01
Aldosterone synthesis and secretion [PATH:ko04925]	8.84E-01	9.10E-01
Focal adhesion [PATH:ko04510]	8.89E-01	9.12E-01
Biosynthesis of unsaturated fatty acids [PATH:ko01040]	8.99E-01	9.21E-01
Vascular smooth muscle contraction [PATH:ko04270]	9.04E-01	9.23E-01
mTOR signaling pathway [PATH:ko04150]	9.26E-01	9.43E-01
Circadian entrainment [PATH:ko04713]	9.33E-01	9.48E-01
Inflammatory mediator regulation of TRP channels [PATH:ko04750]	9.53E-01	9.65E-01
Synaptic vesicle cycle [PATH:ko04721]	9.63E-01	9.73E-01
cGMP-PKG signaling pathway [PATH:ko04022]	9.95E-01	1.00E+00
Collecting duct acid secretion [PATH:ko04966]	9.98E-01	1.00E+00
JAK-STAT signaling pathway [PATH:ko04630]	1.00E+00	1.00E+00
ECM-receptor interaction [PATH:ko04512]	9.97E-01	1.00E+00
Brassinosteroid biosynthesis [PATH:ko00905]	NA	NA
Circadian rhythm - plant [PATH:ko04712]	NA	NA
Hippo signaling pathway - multiple species [PATH:ko04392]	NA	NA
Mitophagy - yeast [PATH:ko04139]	NA	NA
Phototransduction [PATH:ko04744]	NA	NA

Significant differences are shown in bold face (P-value < 0.05).
Abbreviations: KEGG, Kyoto Encyclopedia of Genes and Genomes; FDR, false discovery rate; NA, not applicable.
